# Do health care quality improvement policies work for all? Distributional effects by baseline quality in South Africa

**DOI:** 10.1002/hec.4899

**Published:** 2024-10-03

**Authors:** Finn McGuire, Peter C. Smith, Nicholas Stacey, Ijeoma Edoka, Noemi Kreif

**Affiliations:** ^1^ Centre for Health Economics University of York York UK; ^2^ Imperial College Business School Imperial College London UK; ^3^ SAMRC Centre for Health Economics & Decision Science PRICELESS SA University of Witwatersrand Johannesburg South Africa; ^4^ Health Services and Systems Research Duke‐NUS Medical School Singapore Singapore; ^5^ Faculty of Health Sciences Department of Internal Medicine Health Economics and Epidemiology Research Office University of Witwatersrand Johannesburg South Africa; ^6^ Faculty of Health Sciences School of Public Health University of Witwatersrand Johannesburg South Africa; ^7^ Department of Pharmacy The Comparative Health Outcomes Policy and Economics Institute, University of Washington Seattle USA

**Keywords:** changes‐in‐changes, difference‐in‐difference‐in‐difference, heterogeneous treatment effects, quality improvement

## Abstract

Health care quality improvement (QI) initiatives are being implemented by a number of low‐ and middle‐income countries. However, there is concern that these policies may not reduce, or may even worsen, inequities in access to high‐quality care. Few studies have examined the distributional impact of QI programmes. We study the Ideal Clinic Realization and Maintenance program implemented in health facilities in South Africa, assessing whether the effects of the program are sensitive to previous quality performance. Implementing difference‐in‐difference‐in‐difference and changes‐in‐changes approaches we estimate the effect of the program on quality across the distribution of past facility quality performance. We find that the largest gains are realized by facilities with higher baseline quality, meaning this policy may have led to a worsening of pre‐existing inequity in health care quality. Our study highlights that the full consequences of QI programmes cannot be gauged solely from examination of the mean impact.

## 1 INTRODUCTION

Poor quality has acted to undermine the impact of expansions in the supply and utilization of health care seen in most low‐ and middle‐income countries (LMICs) (Banerjee et al., 2004; Das et al., 2016). Increasing the supply and demand for care without attention to quality may do little to improve health outcomes or protect against the financial risk of health expenditures (Kruk et al., 2018a, 2018b). Further, wide variations in the quality of care provided have been identified in a number of LMICs, often creating and exacerbating health inequalities (Kruk et al., 2017). Just as there are access deserts, quality deserts exist whereby although health care is technically provided, it is not of an adequate standard. This is acknowledged in the WHO concept of “effective coverage” (World Health Organization, 2015). Consequently, many LMICs have attempted to improve health care quality through a range of quality improvement (QI) programmes. QI programmes encompass a wide range of distinct interventions and policies, including public‐private contracting, payment reforms and introducing accreditation standards targeting different components of the quality framework (Rowe et al., 2018, 2019). A specific type of policy we focus on in this paper being national accreditation schemes and supportive supervision (see Section 2). The former are becoming increasingly common in attempts to improve quality standards (Mate et al., 2014), with more than 70 accreditation programmes identified globally in 2013 (Saleh et al., 2013).

Evaluations across a range of QI programmes have found mixed results. Bukonda et al. (2002) noted the positive effect of an accreditation program in Zambia on facilities compliance with outlined standards, but the high associated costs led to the discontinuation of the program. Liberia introduced an accreditation system for all facilities linked to funding eligibility, however large deficiencies in facility standards were identified and follow‐up surveys never completed (Cleveland et al., 2011). A systematic review of the impact of accreditation schemes identified only a modest effect with a median of 7.1% point increase in quality outcomes (Rowe et al., 2018). Bosch‐Capblanch et al. (2011) undertook a review of the impact of managerial supervision to primary health care (PHC) facilities, defined as routine supervision visits of health care providers by higher‐tier or district health workers.^1^ They find nine studies examining various types of managerial supervision schemes across LMICs. The studies looked at a diverse range of outcomes from drug stock management to adherence to standard treatment guidelines with limited evidence of effects found, however the quality of the evidence was deemed to be poor.

These evaluations, typically focusing on a single average treatment effect however, may mask variation in the impact of QI programmes, which is important for two reasons. First, it has been noted that heterogeneity^2^ in the impact of QI programmes—both within and across programmes—may contribute to explaining the mixed results found in evaluations of QI programmes (Binyaruka et al., 2020). A small number of studies have examined heterogenous effects in Performance Based Financing (PBF) schemes in LMICs.^3^ Primarily, differences in effects have been explored across patient sub‐populations (Binyaruka et al., 2018a, 2018b; Lannes et al., 2016; Van de Poel et al., 2015) and facility sub‐groups (Binyaruka et al., 2018a, 2018b; Sherry et al., 2017). Sherry et al. (2017) find heterogeneous responses to a P4P program in Rwanda, specifically effects varying by baseline levels of facility quality, with the largest improvements seen in the medium‐quality tier for both rewarded and unrewarded services. They found high‐quality facilities saw the greatest increase in provision of services with the largest associated financial reward, as they had the highest marginal incentive for doing so. However, this variation is at least partly induced by differential incentives, as program payments were scaled by a general quality multiplier, introducing variation in the incentives for facilities of different baseline quality. In many cases, observed heterogenous policy impacts derive from incentive design effects. Binyaruka et al. (2018a, 2018b) are able to distinguish between incentive design effects and structural effects of a P4P program in Tanzania due to different performance target features used across quality measures. They find the effect of P4P on institutional deliveries, for which facilities face different threshold targets and therefore differential incentives, is largest among baseline low performers, reducing performance inequalities among facilities. For the provision of Intermittent Preventive Treatment for Malaria, for which facilities face identical threshold targets, the effect of P4P was constant across facilities. They note that this goes against much of the literature which predicts failing to account for variation in baseline performance should lead to increases in performance inequality (Rosenthal et al., 2005). Finally, they find that larger facilities and facilities with more supplies received greater P4P pay‐outs. This shows that, while context and program specific factors clearly play a role, examining how the effects of QI programmes vary across units with different baseline characteristics may help understanding the circumstances where QI programmes may be effective. These studies all focused on PBF‐style programmes. To our knowledge, there is no current evidence of heterogeneity in the effects of other forms of QI programmes.

Second, QI programmes have the potential to address distributional concerns by reducing variations in health care quality which contribute to health inequalities. When distributional concerns are important, effect heterogeneity, may have equally important policy implications as average effects. Equality/equity in health facility quality is often a pursued objective due to the concept of horizontal equity, which states that those in equal need to receive equal service, and due to the universalism at the center of the Universal health coverage agenda: “Equitable health services should provide care that does not vary in quality on account of age, sex, gender, race as a social construct, ethnicity or indigeneity, geographical location, religion, socioeconomic status (SES), migrant status, disability, language, sexual orientation, political affiliation or other factors.” (Stevens et al., 2023). However, many studies have identified huge and growing variations in the quality of health care supplied in LMICs (Andrews et al., 2021; Das, 2011; Das & Gertler, 2007; Fink et al., 2019; Haemmerli et al., 2021; Kruk et al., 2017). While there is ample evidence on the inequalities/inequities in quality among health care providers, there has been limited evidence on whether QI programmes have or can reduce this. Consequently, it is important to characterize the distributional impacts of QI programmes: did those at the lower end of the quality distribution gain relatively more from the programmes? As such, standard evaluation methods examining average impacts, implicitly based on fundamental utilitarian principles, may be less appropriate as a normative basis for assessing policy success than evaluations enabling assessment of equity objectives. In addition to equity objectives, there may be efficiency reasons to examine heterogeneity in the effect of QI programmes. Mortality due to the provision of low quality health care remains a significant burden (de Savigny et al., 2004; Kruk et al., 2018a, 2018b). QI programmes could be integral in reducing the health burden attributable to poor quality health care. Despite these important potential benefits, few studies have examined whether QI programmes have contributed to reductions in variations in the quality of health care provided.

In this paper, we examine treatment effect heterogeneity of a QI program—the Ideal Clinic Realization and Maintenance Program (ICRMP)—being implemented in PHC facilities in South Africa (SA). The ICRMP includes two components; a set of quality standards and a QI program to help facilities achieve them. The QI program principally consists of supportive supervision to improve facility functionality and practises. As noted by Dunsch et al. (2022), evaluating a similar type of QI program in Nigeria, improving quality by influencing organizational and managerial practises, without permanently increasing recurrent budgets, would be appealing in many settings but particularly in resource‐constrained LMICs.

Our identification strategy exploits the staggered roll‐out of the ICRMP which led to facilities across a wide range of pre‐treatment quality levels being present in both treated and control group. We employ two primary econometric strategies to identify possible heterogeneous treatment effects. First, we implement a Difference‐in‐Difference‐in‐Difference (DDD) approach enabling identification of different effects of the ICRMP on sub‐groups defined by baseline facility quality levels. We then estimate a Changes‐in‐Changes model which overcomes the issue of any heterogeneity identified being a function of model specification choices, by relaxing some of the DD assumptions, and by explicitly allowing for the identification of distributional impacts (Athey & Imbens, 2006). A previous study of the ICRMP found a significant positive effect of the program on quality checklist scores, while no effect was identified on a range of non‐ICRMP measures (Stacey et al., 2021). However, this average impact may conceal a range of impacts across facilities with important policy implications.

The remainder of the paper is organized as follows. Section 2 provides contextual background and information on the ICRMP. Section 3 summarizes the data. Section 4 presents the methods employed. In Section 5 we present our central results. Section 6 examines the validity of these methods, outlining possible violations in assumptions and assesses the robustness of our results. Sections 7 and 8 provide discussion and conclusion.

## 2 IDEAL CLINIC REALIZATION AND MAINTENANCE PROGRAMME

The ICRMP was established as part of SA's strategy to roll‐out National Health Insurance by 2025. The program was conceived following a 2012 national facility audit, which identified significant short‐comings in the quality of care provided by health facilities in the country. The objective of the program is to set a quality standard for PHC facilities in an attempt to improve health care quality across multiple quality domains.

The ICRMP has two major components; a checklist and a QI program. The ICRMP checklist is a national standardized list of quality indicators against which facilities are assessed and scored, comprised of over 150 indicators separated into 10 components: administration, integrated clinical services management, medicines supplies and laboratory services, human resources, support services, infrastructure, health information management, communication, and stakeholder engagement (Supporting Information S1: Appendix A). Facilities are designated “Ideal” by achieving a weighted average score across indicators, tiered as “vital,” “essential” and “important,” above a single universal threshold value, with scoring undertaken annually. The QI program consists primarily of supportive supervision designed to assist facilities in achieving “Ideal Clinic” status. Supportive supervision entails district‐level Perfect Permanent Teams for Ideal Clinic Realization and Maintenance (PPTICRM) providing assistance to facilities to improve checklist scores with the objective of achieving “Ideal Clinic” status. Facilities are largely expected to improve checklist scores within existing resources budgeted for routinely as part of provincial Health Department budget allocations. However, if deficiencies are identified in facilities' infrastructure or equipment, additional financial resources were provided to QI programmes address this (Hunter et al., 2017).

**FIGURE 1:**
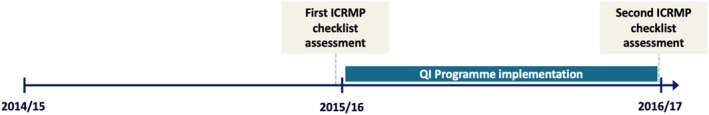
Chronology of ideal clinic realization and maintenance program implementation.

At the start of the 2015/16 Fiscal Year, all PHC facilities in SA undertook an ICRMP checklist assessment. A structured roll‐out of the QI program was planned with facilities allocated years in which they would receive support, with those prioritized starting immediately (2015/2016 FY) (see Figure 1). All PHC facilities were scheduled to receive support over a 3‐year period. Although the ICRMP is a National‐level initiative, the scheduling for facilities' enrollment in the QI program took place at Province‐level by Provincial Departments of Health. However, the initial prioritization of facilities to receive support was largely arbitrary and a systematic process was not followed.

Although all facilities were initially assigned a year for QI program enrollment at the start of 2015/16 FY, some facilities were unenrolled and the scheduled enrollment year of others changed from the start of 2016/17 FY. Therefore, from 2016/17 FY, we cannot rule out facilities' past outcomes impacting their subsequent receipt of support. Consequently, we restrict analysis to the checklist scores corresponding to the start and end of 2015/16 FY. For ease of exposition we refer to the ICRMP assessment taking place at the start of 2015/16 as the 2015 assessment and the one occurring at the end as the 2016 assessment. Additionally, we refer to facilities receiving the ICRMP QI program supportive supervision from April 2015 through to March 2016 (2015/16 FY) as enrolled. Likewise, all facilities not receiving supportive supervision in this period are referred to as non‐enrolled.

There are numerous ways through which the ICRMP QI program may have interacted with pre‐existing health system features, influencing both overall program success and leading to a heterogeneity in benefits.^4^ Specifically, a number of program characteristics provide strong priors for why effect heterogeneity may be found according to baseline quality. All facilities enrolled into the program are provided with additional financial resources, where required, for QI while the nature of the ICRMP quality indicators is such that population characteristics and demand‐side factors should not play a strong determining role in Quality scores (QS). As such, whereas heterogeneous treatment effects observed in previous studies may stem from differences in marginal costs of quality improvement or differential incentive sizes, in the SA context these should be largely constant across QI program recipients. Assuming that quality is a function of capacity and effort, pre‐existing quality variation may be caused by variation in facility capacity or facility staff efforts. If capacity is the constraint, we would expect that we may see a greater treatment effect among low baseline performers due to the QI program provision of guidance materials, support and additional financial resources to meet deficiencies in infrastructure and equipment. However, if pre‐existing quality variation is caused by differential effort, then we might expect a larger treatment effect among high baseline performers. Therefore, the ICRMP's idiosyncratic features suggest heterogeneous treatment effects according to baseline performance may be expected. In addition heterogeneity identified may potentially reveal information on the factors determining variation in facility quality, although data constraints prevent a comprehensive investigation of mechanisms.

## 3 DATA

Our primary dataset is the ICRMP data collected during routine assessments. This facility‐level data provides information on ICRMP QI program enrollment and ICRMP checklist score information. Table 1 shows that 3433 PHC facilities were in operation across the nine Provinces of SA in this period. All Provinces—with the exception of Western Cape—had PHC facilities that were both enrolled and not enrolled in the ICRMP during the 2015/16 FY.

**TABLE 1:** PHC facilities and QI enrollment by province.

Province	Number of PHC facilities	Proportion of total PHC facilities (%)	Not enrolled in QI 2015/16	Enrolled in QI 2015/16	Proportion of PHCs enrolled (%)
Eastern cape	763	22	529	234	31
Free state	221	6	121	100	45
Gauteng	370	11	195	175	47
Kwazulu‐natal	597	17	394	203	34
Limpopo	473	14	300	173	37
Mpumalanga	284	8	198	86	30
North west	305	9	203	102	33
Northern cape	160	5	101	59	37
Western cape	260	8	260	0	0
Total/average	**3433**	**100**	**2301**	**1132**	**33**

The diverse range of ICRMP components provide an overview of structural and process indicators of PHC quality (Donabedian, 1988). The assessments share many characteristics with globally undertaken Service Availability and Readiness Assessments (WHO) and Service Provision Assessments (USAID). Our primary outcome variable is the aggregated ICRMP checklist scores. This provides a composite quality index between 0 and 100 symbolizing the percentage of ICRMP quality indicators each facility has satisfied, providing a measure of facilities' ability to deliver quality health care. ICRMP checklist score data is available on 2381 facilities.^5^

Appendix Figure S1a,b (Supporting Information S1: Appendix B) show the full distributions of the ICRMP QS by baseline quality quartiles for the 2015 and 2016 assessments respectively. They show a high degree of variation in changes in ICRMP QS both across and within baseline stratum.

In addition to the ICRMP‐specific data we utilize a number of other data sources. The District Health Information System (DHIS) routinely compiles monthly facility‐level activity data. For our purposes we primarily use a measure of monthly patient headcount and a measure of facility labor supply; number of clinic nurse work days per month. Both are aggregated to provide annual counts. The South Africa Index of Multiple Deprivation provides socio‐demographic characteristics of areas surrounding health facilities (Noble et al., 2013). Finally, we use spatial population distribution data from Afripop (Linard et al., 2012) to create population densities surrounding health facilities.^6^

Table 2 presents descriptive statistics comparing enrolled and non‐enrolled facilities for the full sample as well as within baseline quality strata. While facility and local‐area level characteristics are mostly similar across enrolled and non‐enrolled, there are a number of characteristics where enrolled and unenrolled facilities appear to have slight differences. For the total sample of facilities, those enrolled in the QI program have 23% more monthly professional nurse clinic working days than unenrolled clinics (103 vs. 84 days), and 30% higher patient headcounts. We use an *F*‐test to check the joint hypothesis that the variables are balanced between enrolled and unenrolled facilities. The test confirms that enrolled and unenrolled facilities are not statistically balanced in observed variables.

**TABLE 2:** Descriptives statistics.

	All facilities		Lowest base Q		Low base Q		High base Q		Highest base Q	
	QI enrolled facilities	Non‐enrolled facilities	QI enrolled facilities	Non‐enrolled facilities	QI enrolled facilities	Non‐enrolled facilities	QI enrolled facilities	Non‐enrolled facilities	QI enrolled facilities	Non‐enrolled facilities
Municipal socio‐demographics										
Population <15 years (,000)	202 (282)	194 (275)	211 (299)	178 (271)	177 (256)	182 (270)	199 (282)	208 (279)	221 (291)	212 (281)
Population >60 (,000)	57 (82)	54 (81)	60 (87)	49 (80)	50 (75)	51 (80)	56 (82)	58 (82)	61 (83)	59 (83)
Household size	3.4 (0.5)	3.5 (0.5)	3.4 (0.4)	3.5 (0.5)	3.4 (0.4)	3.5 (0.5)	3.4 (0.5)	3.5 (0.4)	3.5 (0.5)	3.5 (0.5)
Proportion with no schooling	0.09 (0.04)	0.09 (0.04)	0.09 (0.05)	0.10 (0.05)	0.09 (0.04)	0.09 (0.04)	0.09 (0.04)	0.09 (0.04)	0.10 (0.04)	0.09 (0.04)
Proportion population with primary education	0.06 (0.01)	0.06 (0.01)	0.06 (0.01)	0.06 (0.01)	0.06 (0.01)	0.06 (0.01)	0.06 (0.01)	0.06 (0.01)	0.06 (0.01)	0.06 (0.01)
Proportion population with secondary education	0.18 (0.06)	0.17 (0.06)	0.17 (0.07)	0.17 (0.06)	0.17 (0.06)	0.18 (0.06)	0.18 (0.06)	0.19 (0.06)	0.18 (0.06)	0.18 (0.06)
Proportion with no income	0.41 (0.03)	0.41 (0.03)	0.42 (0.03)	0.42 (0.03)	0.41 (0.03)	0.42 (0.03)	0.41 (0.03)	0.42 (0.03)	0.42 (0.03)	0.42 (0.03)
Proportion of population black	0.86 (0.17)	0.88 (0.17)	0.86 (0.19)	0.86 (0.22)	0.85 (0.20)	0.88 (0.16)	0.86 (0.17)	0.89 (0.14)	0.88 (0.15)	0.91 (0.11)
Proportion population urban dwelling	0.49 (0.39)	0.43 (0.37)	0.49 (0.39)	0.42 (0.37)	0.50 (0.38)	0.46 (0.38)	0.52 (0.38)	0.44 (0.37)	0.46 (0.39)	0.38 (0.36)
Proportion households with flush toilet	0.45 (0.32)	0.41 (0.30)	0.45 (0.33)	0.40 (0.30)	0.46 (0.32)	0.44 (0.31)	0.47 (0.32)	0.41 (0.30)	0.43 (0.31)	0.37 (0.29)
Proportion households with piped water	0.63 (0.26)	0.62 (0.24)	0.64 (0.26)	0.63 (0.25)	0.64 (0.27)	0.65 (0.24)	0.64 (0.27)	0.63 (0.23)	0.62 (0.26)	0.57 (0.23)
PHC Local geography										
Distance to closest PHC (km)	6.3 (8.2)	6.2 (8.6)	5.1 (5.0)	6.8 (10.0)	6.4 (8.4)	5.7 (8.3)	6.6 (9.6)	6.3 (9.4)	6.7 (8.2)	5.7 (5.1)
Number of PHCs in 10 km	6.2 (7.8)	5.4 (7.0)	7.6 (9.7)	5.2 (7.4)	5.9 (7.6)	5.4 (6.8)	5.8 (7.2)	5.6 (6.9)	5.8 (7.2)	5.5 (6.9)
Population within 10 km	189,314 (339,965)	170,135 (309,225)	234,052 (421,229)	170,655 (333,963)	169,057 (334,803)	162,653 (299,313)	170,583 (303,540)	169,475 (290,557)	195,905 (317,867)	179,006 (304,656)
Number of PHCs in local municipality	23 (18)	26 (22)	26 (20)	26 (24)	25 (18)	23 (20)	24 (20)	27 (23)	20 (14)	25 (22)
DHIS										
Monthly professional nurse clinic working days	103 (102)	84 (95)	102 (90)	74 (70)	98 (97)	80 (72)	107 (118)	93 (144)	105 (97)	90 (80)
Monthly patient headcount	3373 (3287)	2594 (2366)	3221 (2968)	2298 (1918)	3038 (2683)	2530 (2230)	3544 (3833)	2634 (2347)	3588 (3373)	3045 (2980)
Monthly new fully immunized <1 year	22.8 (22.8)	18.8 (17.7)	21.5 (22.5)	18.0 (18.3)	20.8 (19.8)	17.9 (14.8)	23.4 (23.6)	19.2 (18.4)	24.8 (24.6)	20.3 (19.0)
Monthly antenatal 1st visit before 20 weeks (%)	64.9 (11.7)	65.2 (11.4)	63.9 (12.0)	64.4 (11.5)	64.7 (12.2)	65.1 (11.3)	65.5 (11.5)	65.3 (11.9)	65.3 (11.3)	66.6 (10.9)
Monthly cervical cancer screening	19.4 (20.1)	16.1 (16.6)	16.9 (14.7)	14.3 (14.3)	17.7 (17.5)	16.8 (19.3)	21.0 (23.3)	16.2 (15.9)	21.0 (21.4)	17.9 (16.9)
Monthly HIV positive new eligible client initiated on IPT	11.3 (12.6)	10.6 (15.5)	9.9 (9.4)	9.4 (12.9)	9.7 (9.8)	10.1 (12.8)	12.2 (14.9)	10.6 (15.5)	12.7 (13.8)	13.0 (20.6)
Monthly tracer item stock out rate	25.0 (30.7)	22.9 (29.9)	24.7 (30.8)	23.1 (31.3)	27.4 (32.1)	23.6 (29.8)	25.3 (30.9)	25.5 (31.1)	22.8 (29.1)	18.7 (26.2)
South African index of multiple deprivation										
SES 1st quantile	0.06	0.05	0.03	0.08	0.07	0.05	0.09	0.03	0.05	0.02
SES 2nd quantile	0.09	0.08	0.10	0.08	0.09	0.08	0.10	0.09	0.08	0.07
SES 3rd quantile	0.13	0.13	0.14	0.12	0.10	0.14	0.13	0.12	0.15	0.15
SES 4th quantile	0.23	0.28	0.21	0.24	0.23	0.29	0.19	0.28	0.27	0.34
SES 5th quantile	0.49	0.46	0.52	0.48	0.51	0.44	0.48	0.49	0.45	0.42
Balance test (*F*‐test for joint orthogonality)	3.50***		2.46***		2.38***		2.32***		2.08***	

*Note*: Main statistics reflect means or proportions. Standard deviations reported in parentheses. F‐statistic tests joint hypothesis of all characteristics being equal to 0 in determining treatment status in the baseline year that is, 2015.

Abbreviation: SES, socio‐economic status.

*p < 0.1, **p < 0.05, ***p < 0.01.

We also check whether there are differences in baseline QS between facilities of different “sizes.” Although larger facilities—as measured by average monthly patient headcount and number of nurse working days—have slightly higher baseline QS, the magnitude of this difference is not large (Supporting Information S1: Appendix C).

## 4 METHODS

### 4.1 Difference‐in‐difference‐in‐difference

We first estimate the effect of the QI program on facilities of differing pre‐treatment quality within a DDD framework. DDD has been used to improve the validity of standard DD models (Long et al., 2010; Rosenbaum, 1987; Yelowitz, 1995), but can also allow the estimation of sub‐group effects of a treatment (Stokes et al., 2017). We estimate both stratified regression models across the sub‐samples defined by baseline quality and, to preserves the full statistical power of the available sample (Wang & Ware, 2013), a single regression allowing interactions between treatment and baseline quality strata. Identification in both approaches assumes that after controlling for facility‐level covariates and fixed unobserved effects, QI program assignment can be considered random. There is no evidence of geographical clustering of ICRMP enrollment or baseline quality levels (see Supporting Information S1: Appendix D). This restricts concern to facility‐level time‐varying heterogeneity. So there may be unobservable time‐varying differences between facilities who are enrolled in the QI program and those that aren't^7^.

We estimate regression models for each of the strata defined by baseline quality *g* as:

(1) $$Y_{fgt}=\alpha_{fg}+\gamma_{g}{QI}_{fgt}+\eta_{gt}+\beta_{g}X_{fgt}+\varepsilon_{fgt}$$

Where *f* = 1,…,*F* indexes facilities, and *t* = 1,…,*T* indexes time periods. We split the sample of facilities four quartiles, *g* = 1,2,3,4, corresponding to the quartiles of baseline quality. As enrollment occurs at facility‐level we have enrolled and non‐enrolled facilities within each stratum. *Y*_*fgt*_ represents the aggregate ICRMP quality score for each facility *f* part of strata *g* for every period *t*; *QI*_*fgt*_ denotes enrollment in the ICRMP QI program; *γ*_*g*_ is the treatment effect of interest for a given group *g*, ***X***_*fgt*_ represents a vector of time‐varying facility‐ and small area‐level covariates that may affect ICRMP quality score attainment and enrollment in the QI program. *α*_*fg*_ are time‐invariant facility‐specific omitted factors impacting QS. We also include *η*_*gt*_ to capture the general secular trend in the ICRMP QS, controlling for unobserved variables that evolve over time across all facilities. This may capture differences in information about the calculation of QS facilities are given across years. These models allow the effect of all the control variables on the outcome to differ by baseline quality.

For the interaction model, we estimate:

(2) $$Y_{ft}=\alpha_{f}+\gamma {QI}_{ft}+\sum_{g=2}^{4}\gamma_{g}^{\prime }\left({BaseQul}_{f}*{QI}_{ft}\right)+\eta_{t}+\sum_{g=2}^{4}\phi_{g}\left({BaseQul}_{f}*\eta_{t}\right)+\beta X_{ft}+\varepsilon_{ft}$$

All variables remain the same as (1) but instead of estimating over *g* samples, we include *BaseQul*_*f*_ representing facilities' pre‐enrollment ICRMP quality score. *BaseQul*_*f*_ is time‐invariant and therefore its main effect cannot be estimated by FE, but we can estimate it's interaction the effect with the QI program enrollment, on facility QS.^8^ *γ* is the treatment effect for the lowest baseline quality strata. $\gamma_{g}^{\prime }$ is then a second‐order interaction outlining the difference in the effect of the QI program for quality strata relative to the effect on the lowest baseline strata. We avoid the common strong assumption of linear interaction effects in regression‐based multiplicative interaction models by estimating separate parameters for the effect of the QI program for each baseline quality quartile (Hainmueller et al., 2019).^9^, ^10^ Finally, we allow for differential time trends in the outcome by baseline quality strata as implied by Table 2.^11^ Identification via DDD takes the change in the non‐enrolled facilities as the counterfactual change for enrolled facilities. Therefore, allowing for differential time trends between strata allows unobserved variables that evolve over time to differ by strata of baseline quality. For instance, facility‐level funding changes over time may be related to relative quality levels rather than a constant change across all facilities. However, this specification still requires that the counterfactual trends of facilities enrolled in the QI program and non‐enrolled facilities within the same quality strata are parallel. We also estimate a pooled ordinary least squares (POLS) version of equation (2) to see the impact of including facility fixed effects.

An important distinction should be made between our models and lagged dependent variable (LDV) specifications.^12^ Although we include the outcome as an explanatory variable, a key distinction is that baseline quality is time‐invariant. Consequently, the models do not suffer from Nickell bias associated with dynamic models with fixed effects (Nickell, 1981) (See Supporting Information S1: Appendix F for detail). Because only facilities whose treatment status changes contribute to the estimation of the treatment effects, *γ* and $\gamma_{g}^{\prime }$, our estimates provide the average treatment effect on the treated (ATT) for each sub‐group. The model assumes no heterogeneity in the effects of the covariates included in the model but not interacted. Our DDD model, therefore, does not estimate full conditional average treatment effects on the treated (CATTs) as they do not allow the effects of all covariates to vary across the four strata (Gibbons et al., 2019).

Two sets of confounding factors must be considered to make causal claims with interaction analysis; treatment‐outcome and moderator‐outcome confounders (VanderWeele, 2015). However, if only potential confounding factors of the relationship between enrollment in the QI program and the ICRMP checklist score are considered, this is sufficient for identifying effect heterogeneity across the strata examined. The coefficients on the interaction terms can be considered the causal effects of the QI program within each stratum defined by baseline quality score and the differences a measure of heterogeneity in this causal effect. We control for facility's patient headcount and a measure of facility staffing (nurse working days) as Table 2 revealed differences between enrolled and non‐enrolled facilities in facility size by these metrics. It is possible that facility size, patient volume, staffing levels and related activity intensity may influence the ability of facilities to attend to quality deficiencies highlighted in the ICRMP assessments.

### 4.2 Changes‐in‐changes

A limitation with the above approach is the counterfactuals, and therefore treatment effects identified, are functions of how subgroups are composed that is, treatment effect heterogeneity is a parametric function of the number of groups, allowing identification of $E\left[Y_{i}^{T}-Y_{i}^{C}|g\right]=\gamma_{g}$ with constant treatment effect within these groups imposed. Therefore, while the DDD estimates give an indication of the pattern of treatment effect heterogeneity, it cannot identify the treatment effect of the QI program across the full distribution of QS in our data. The DDD model also relies on strong linear additive separability assumptions. Although our DDD models allow the impact of time and treatment effect to vary, this is done in a restricted way, across strata of pre‐treatment quality. Additionally, additivity assumptions imply that facility returns to the QI program are not affected by unobserved facility characteristics such as staff effort or managerial quality. Further, the model still implies additive separability between the treatment and unobservables. Therefore, we still require conditional mean independence of the unobservables and enrollment status within pre‐treatment strata.

To avoid the impact of modeling specifications on heterogeneity identified and relax the additive separability assumptions, we implement the Changes‐in‐Changes (CC) model proposed by Athey and Imbens (2006). With CC, we are able to estimate the full counterfactual distribution of QS that QI enrolled facilities would have achieved if they had not been enrolled. The CC model is based on a single non‐separable equation allowing for arbitrary interactions between treatment and unobservable characteristics through a structural function *h*(.). This allows the distribution of unobservables to be arbitrarily different across enrolled and non‐enrolled facilities that is, *F*_*U*_|*QI* = 1 does not need to be the same as *F*_*U*_|*QI* = 0.

The intuition for identification and estimation for the CC model is similar to DD, with some important distinctions. Estimation of the CC model requires comparing the quality score cumulative distribution functions (CDFs) of the four treatment‐by‐period groups, rather than just the first moments. The change in the distribution of the QS for unenrolled facilities over 2015/16 is used to estimate the counterfactual CDF of the QS enrolled facilities would have achieved over the same period had they not been enrolled, $F_{Y_{1}^{T}\left(0\right)}$. Specifically, $F_{Y_{1}^{T}\left(0\right)}$ is identified by (Athey & Imbens, 2006):

$$F_{Y_{1}^{T}\left(0\right)}\left(Y\right)=F_{Y_{0}^{T}}\left(F_{Y_{0}^{C}}^{-1}\left(F_{Y_{1}^{C}}\left(Y\right)\right)\right)$$

Where $F_{Y_{0}^{T}}$ is distribution of the pre‐treated outcomes for the treated group, $F_{Y_{0}^{C}}^{-1}$ is the inverse of the distribution of the pre‐treated outcomes for the control group and $F_{Y_{1}^{C}}$ is the distribution of the post‐treatment outcomes for the control group. As the three distributions on the RHS are observable, the LHS is identified.

Having constructed the full counterfactual distribution of QS, we can estimate the full set of quantile treatment effect on the treated (QTT). For example, the QTT for the 20^th^ percentile is the difference in the potential outcome distributions for enrolled facilities at the 20^th^ percentile of the quality score distribution without QI enrollment. Consequently, the QTT at each quantile *q* is calculated as the difference between the inverse of the constructed counterfactual CDF, $F_{Y_{1}^{T}\left(0\right)}\left(Y\right)$, and the inverse of the observed CDF for the enrolled facilities post‐treatment:

(3) $$QTT\left(q\right)=F_{Y_{1}^{T}\left(1\right)}^{-1}\left(q\right)-F_{Y_{1}^{T}\left(0\right)}^{-1}\left(q\right)$$

While the ATTs given by the DDD provide treatment effect estimates for clearly defined sub‐groups, CC identifies treatment effect heterogeneity across quantiles of the counterfactual outcome distributions.^13^ Borrowing a phrase from Djebbari and Smith (2008) the QTT reflects “impacts *at* quantiles rather than *on* quantiles.”

The key assumptions underlying construction of the counterfactual distribution are largely generalizations of DD assumptions. QS, are assumed to be generated by an unknown non‐separable function; *QS* = *h*(*U*,*T*). Where *U* is a vector capturing unobservable facility characteristics and *T* is time. As *QS* does not depend on enrollment status, enrolled and non‐enrolled facilities in the pre‐treatment period with the same quality score, *QS* ^′^, must have identical *U* = *u*. There are two primary assumption difference between DD and CC (see Supporting Information S1: Appendix G for full technical assumptions). CC requires the function *QS* = *h*(*U*,*T*) be strictly monotone increasing, that is, ∆*h*(*U*) > 0, so higher unobservables result in strictly higher outcomes. This is non‐restrictive in our case as it is natural to assume that greater effort or capacity result in higher QS. Additionally, the distribution of unobservable facility characteristics are time invariant within both enrolled and non‐enrolled groups, *U*⊥*T*|*QI*. As such, QS may change over time through the previously outlined production function, *QS* = *h*(*U*,*T*), but because the within group distribution of *U* is time‐invariant, the change can only reflect a time effect. Therefore, non‐enrolled facilities at the same quantile *q* of their respective outcome distribution pre‐, $F_{Y_{0}^{C}}$, and post‐treatment, $F_{Y_{1}^{C}}$, may have different outcomes due to an effect of *T* but must have identical *U* = *u*. Therefore the evolution in outcomes for non‐enrolled facilities at quantile *q* provides a counterfactual for the evolution in outcomes for enrolled facilities with pre‐treatment quality scores, *QS* ^′^, had these facilities not been enrolled. The QTT can then be calculated for the full support of QS of enrolled facilities post‐enrollment.

Although pre‐treatment we may have $F_{Y_{0}^{T}}\neq F_{Y_{0}^{C}}$ due to $F_{U_{T}}\neq F_{U_{C}}$, the assumed time‐variance of the distribution of unobservables—$F_{U_{T0}}=F_{U_{T1}}$ and $F_{U_{C0}}=F_{U_{C1}}$—means, in the absence of treatment, both groups would have seen the same growth in quality score; *QS* = *h*(.,*T*). Consequently, unlike DD frameworks, the CC identifying assumption is invariant to transformations of the outcome variable as common growth in the outcome is assumed rather than parallel trends (Lechner, 2011). Specifically, the assumption is that the change in QS over 2015/16 is the same for facilities with pre‐treatment quality *QS* ^′^ in both enrolled and non‐enrolled groups, in the absence of the QI program.

Not including relevant time‐varying covariates can cause differences in the production functions between the enrolled and non‐enrolled groups that map the unobservables to outcomes in a given period, and would lead to inconsistent estimates of QTTs. Following Melly and Santangelo (2015), we use an extension to the CC model allowing the incorporation of covariates. This estimation approach is based on a semi‐parametric quantile regression with identification of the counterfactual distribution following from the Athey and Imbens (2006) approach described above (Supporting Information S1: Appendix H).^14^ We estimate the near full set of QTTs, resulting in 99 QTTs.

## 5 RESULTS

### 5.1 Difference‐in‐difference‐in‐difference

Table 3 presents average facility QS by baseline quartiles, illustrating the large pre‐existing variation in scores. In 2015, the average score for facilities in the top quartile of the baseline quality score distribution was 79% higher than the average score of facilities in the bottom quartile. However, average quality over the period converges across facilities with different baseline scores, with the average score of facilities in the bottom 25th percentile at baseline increasing over 15 points by 2016 while scores of facilities in the 75^th^ percentile decreased.

**TABLE 3:** ICRMP checklist scores trends.

	Lowest baseline quality facilities	Low baseline quality facilities	High baseline quality facilities	Highest baseline quality facilities	All facilities
Average (SD) aggregate quality 2015	40.1 (6.0)	51.7 (2.4)	59.8 (2.4)	71.8 (6.2)	55.9 (12.5)
Average (SD) aggregate quality 2016	55.7 (14.6)	59.9 (13.9)	63.0 (16.1)	67.5 (17.3)	61.5 (16.1)
Average aggregate score change	15.6	8.2	3.2	−4.3	5.6
Observations	596	595	595	595	2381

*Note*: SD is standard deviation of the mean scores.

Table 3 also reassures that we do not need to be concerned about potential ceiling effects on further quality improvements for the highest baseline performers.

In lieu of data to examine pre‐treatment trends in ICRMP QS for enrolled and non‐enrolled facilities, we note the similarities in pre‐treatment levels of the scores and facility characteristics (Supporting Information S1: Appendix I). The largest within quartile pre‐treatment difference in average QS between enrolled and non‐enrolled facilities is 1.6, for the lowest baseline performing quartile. Additionally, the distributions of within quartile QS are almost identical.

Tables 4 and 5 present the results of Equations (1) and (2) respectively. For all specifications, we clustered our standard errors at the facility‐level to address concerns of serial correlation (Bertrand et al., 2004). The estimated coefficients capturing the effect of the QI program for the stratified and interaction regressions are, as expected, almost identical.^15^ From Table 4 we see that the effect of the QI program is positive and significantly different from 0 for all baseline quality strata. The impact of the QI program increases with the facilities' baseline quality. The effects represent 29% of the average score for the highest performing and 15% of lowest performing strata at baseline (Table 3), showing the estimated treatment effects are regressive even from a proportional perspective. The POLS and FE estimates are almost identical (Table 5), indicating that controlling for facility fixed effects appears to have little impact on effect estimates.

**TABLE 4:** FE stratified regressions.

	All facilities	Lowest baseline quality	Low baseline quality	High baseline quality	Highest baseline quality
QI program	10.06*** (0.668)	6.103*** (1.318)	6.596*** (1.115)	15.77*** (1.153)	20.75*** (1.190)
Year	1.092* (0.504)	13.70*** (0.730)	5.066*** (0.753)	−4.327*** (0.834)	−15.62*** (0.951)
R^2	0.175	0.539	0.318	0.27	0.394
F	170.3	173.9	74.61	60.18	84.09
Observations	4727	1178	1181	1181	1187

**TABLE 5:** Multiplicative interaction models.

	POLS	FE
QI prog lowest baseline quality (γ)	7.702*** (1.197)	6.064*** (1.312)
QI prog low baseline quality relative to *γ*	−0.251 (1.592)	0.651 (1.722)
QI prog high baseline quality relative to *γ*	7.665*** (1.612)	9.671*** (1.747)
QI prog highest baseline quality relative to *γ*	12.21*** (1.578)	14.74*** (1.769)
Year lowest baseline quality	13.21*** (0.712)	13.57*** (0.734)
Year low baseline quality	−8.204*** (1.012)	−8.378*** (1.044)
Year high baseline quality	−17.27*** (1.073)	−17.99*** (1.109)
Year highest baseline quality	−28.10*** (1.122)	−29.10*** (1.202)
R^2	0.547	0.403
F	358.3	151.5
Observations	4727	4727

Abbreviation: POLS, pooled ordinary least squares.

Notably, there are vastly differential time trends, as for facilities with lowest/low baseline quality, there is a general increase in the quality score over time, while the opposite is true for facilities with high/highest baseline quality. This trend among untreated facilities suggests facility quality may be subject to noisy measurement, which we seek to address in the sensitivity and elaborate on in the discussion. The estimated time trend is small when examining all facilities together (Table 4). Subsequently, the ratio of the effect of the QI program and the time effect would lead to the conclusion that the QI program has an exceptional effect size. However, once the time trend is allowed to vary by baseline quality it becomes clear that the small average common trend is composed of large and opposing trends across facility strata types. It is clear the time effect parameter is large in absolute size within these strata. This suggests sizable over time changes in QS may not be uncommon and that more evidence on the variance of intra‐facility quality may be beneficial.

### 5.2 Changes‐in‐changes

**FIGURE 2:**
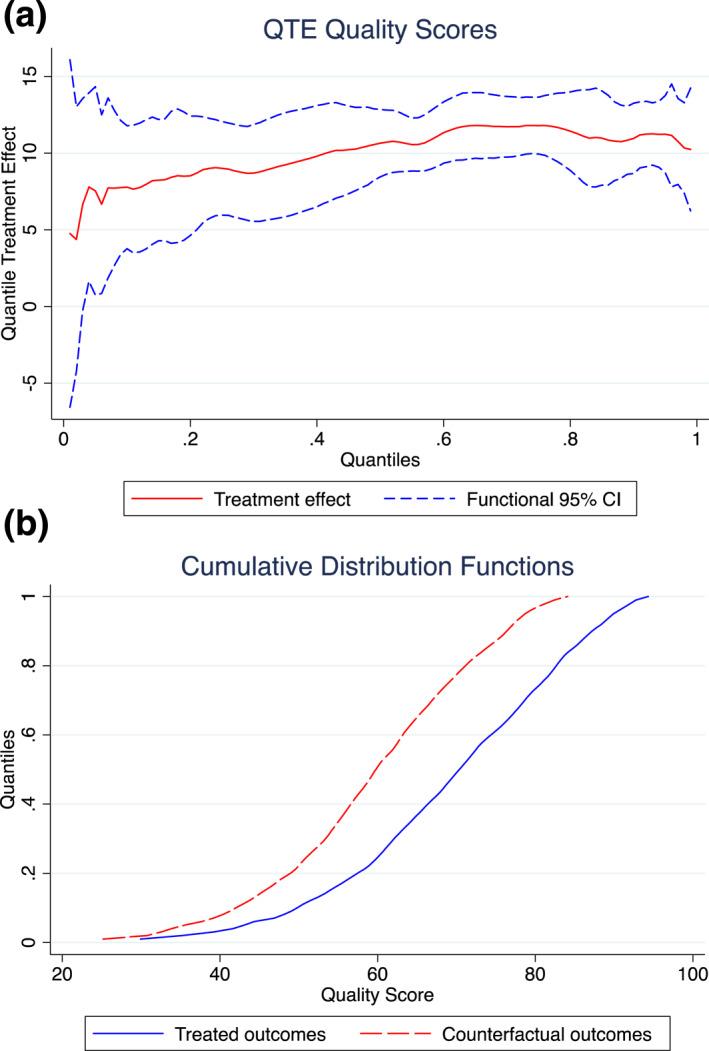
(a) CC model with covariates. and (b) show results of CC model with covariates.

Figures 2a,b presents results of the CC model with covariates,^16^ evaluating the QTT at every percentile point of the distribution (without covariates presented in Supporting Information S1: Appendix J).^17^ The estimated effect of the QI program is positive across the whole of the quality score distribution, with the estimated confidence bands including zero only for the lowest fraction of the distribution. The pattern of treatment effects reflects that of the DDD results, with the effect of the QI program increasing along the distribution of pre‐treatment QS. Including covariates reduces the range of QTTs observed from 2.4—12.6 to 4.4–11.8. Despite this marginal impact on the point estimates, in the model with covariates we cannot reject the hypothesis that all QTTs are equal to the median QTT, whereas without covariates this was rejected at the 1% level. Accompanying tables for the CC figures and a direct comparison of the QTTs with and without covariates is found in Supporting Information S1: Appendix K. All figures include 90% confidence bands based on a non‐parametric bootstrap with 1000 replications.

Although not directly comparable, the 90% confidence bands from the CC estimates include the point estimates of the treatment effect for all but the final stratum from the DDD results. However, due to the different estimands examined this restricts the value in making direct effect comparisons across the methods.

### 5.3 Impact of ICRMP QI program on DHIS2 measures

We also examine whether there are heterogeneous treatment effects on a number of indicators from the DHIS 2 (DHIS2). Specifically, following Stacey et al. (2021) we examine whether the QI program has an impact on Early antenatal visit rate (%); HIV positive clients initiated on isoniazid preventive therapy (N); Infant patients fully immunized (N) and; Cervical cancer screening for women over 30 (N). We find the QI program has no significant effect on the four measures across facilities for all baseline quality quartiles. Detailed results on these analyses are presented in Supporting Information S1: Appendix L.

The findings of null effects for these measures, principally capturing the quantity of services delivered is unsurprising. The hypothesis that the QI program would increase or improve these measures relies on the assumption that patterns of patient demand respond to changes in facility quality. For patient demand to respond to changes in facility quality two conditions are required. First, patients must have sufficient information on the quality of their choice set of health facilities, enabling informed choice and the adaptation of choices to changes in provider characteristics. Second, patients must face a low cost of switching between health facilities. Without a low switching cost, the quality changes health facilities must undertake to induce patient switching increases. It has been shown that patients in LMICs lack sufficient information to allow informed utilization choices (Pickett, 2016).

## 6 SENSITIVITY CHECKS

Policy endogeneity may undermine the ability to exploit policy variation for identification (Besley & Case, 2002). One possibility is that QI program enrollment may have been influenced by views on the performance of facilities. Facilities viewed as recent past performance improving may have been prioritized for enrollment in the program, partly to encourage continued quality gains. If already improving facilities are enrolled in the QI program, this will upward bias the estimated effects of the program. Zeldow and Hatfield (2021) illustrate how the parallel trends assumption can be violated if 1) the unobserved fixed effects are not balanced and the effects of the unobserved fixed effects are not constant or 2) there are unobserved time‐varying confounders with differentially evolving trends or effects. In our setting, if there is a prioritization of facilities based on recent performance improvements, resulting from higher effort levels or capacities, this will result in unobserved differences between enrolled and unenrolled facilities. Below we examine three approaches that allow for, control or reveal the potential for self‐selection and ensuing unobserved differences between enrolled and unenrolled facilities.

### 6.1 Lagged dependent variable model

Due to these questions around the parallel trend assumption, we estimate a LDV model which allow the effects of unobserved confounders to change over time (Ding & Li, 2019; O’Neil et al., 2016). LDV models assume selection based on past outcomes and, therefore, imply unconfoundedness conditional on the lagged values of the outcome:

$$Y_{1}^{T}\left(0\right),Y_{1}^{C}\left(0\right)⊥{QI}_{f}|Y_{f0},X_{ft}$$

If it is the case that enrollment in the QI program is determined by lagged dependent variables then fixed effects estimates are not consistent (Angrist & Krueger, 2000). We run an OLS which controls for the lagged outcome level and as before, allows for differential policy effects in the four strata based on pre‐policy quality score quartiles:

$$Y_{ft}=\alpha +\sum_{g=1}^{4}\gamma_{g}\left({BaseQul}_{f}*{QI}_{ft}\right)+\theta Y_{ft-1}+\beta X_{ft}$$

The estimated effects from the LDV models are very similar to those of the DDD models (Table 6).

**TABLE 6:** Lagged dependent variable.

	(1)	(2)	(3)	(4)
QI prog lowest baseline quality (γ)	7.432***	6.818***	6.618***	7.559***
	(1.226)	(1.158)	(1.159)	(1.150)
QI prog low baseline quality relative to *γ*	−0.481	0.0279	0.157	−0.239
	(1.676)	(1.582)	(1.580)	(1.563)
QI prog high baseline quality relative to *γ*	8.217***	8.009***	7.996***	7.627***
	(1.672)	(1.580)	(1.579)	(1.561)
QI prog highest baseline quality relative to *γ*	13.25***	12.99***	13.06***	12.21***
	(1.672)	(1.578)	(1.575)	(1.559)
Lagged ICRMP quality score	0.289***	0.249***	0.248***	0.256***
	(0.0614)	(0.0581)	(0.0580)	(0.0573)
Proportion population no education		13.08	13.62	7.420
		(10.36)	(10.38)	(10.39)
Proportion population no income		−45.41***	−48.95***	−44.58***
		(10.34)	(10.37)	(10.34)
Proportion population urban		4.540	4.538	4.107
		(3.115)	(3.141)	(3.179)
Household size		7.239***	7.296***	7.371***
		(0.886)	(0.889)	(0.920)
Proportion households with flush/Chemical toilet access		22.50***	21.82***	19.26***
		(3.936)	(3.974)	(3.998)
Proportion households with piped/Borehole water access		−16.75***	−16.69***	−14.61***
		(2.170)	(2.176)	(2.182)
SES quintile		1.491***	1.302***	1.508***
		(0.267)	(0.278)	(0.297)
Mean monthly nurse working days			0.00574*	0.00610*
			(0.00321)	(0.00317)
Mean monthly patient headcount			0.000106	−0.0000688
			(0.000134)	(0.000138)
Distance to closest other PHC facility (km)				0.0363
				(0.0354)
Number of PHC facilities within 10 km				−0.766***
				(0.108)
Population within 10 km				0.0000189***
				(0.00000252)
District				0.0326
				(0.0199)
Constant	41.76***	29.60***	31.19***	28.52***
	(2.524)	(5.796)	(5.807)	(5.785)
R‐sq	0.264	0.349	0.352	0.370
F	106.5	84.26	75.00	65.52
N	2381	2373	2365	2365

*Note*: Standard errors in parentheses, *p < 0.1, **p < 0.05, ***p < 0.01.

Abbreviation: SES, socio‐economic status.

### 6.2 Matching on pre‐treatment quality performance

However, as we only have outcome data for one pre‐treatment period available, this restricts our ability to examine and condition on past outcomes and doesn't provide evidence to examine if enrolled facilities were experiencing a pre‐treatment increase in quality.^18^, ^19^, ^20^ In order to gain more insight into pre‐treatment facility performance we utilize DHIS data on facility activity. This represents the most comprehensive formal data on facility performance prior to the implementation of the ICRMP checklist. Table 7 outlines the DHIS facility activity variables.

**TABLE 7:** DHIS variables (data from January 2013—June 2015).

Monthly children <1 year fully immunized
Monthly patient head count
Monthly patients seen by professional nurse
Monthly professional nurse days at facility
Monthly rate of ANC 1st visit before 20 weeks
Monthly number of cervical cancer screenings >30 years
Monthly number of measles 1st dose
Monthly number of RV 2nd doses for <1 year
Monthly number of HIV + new client initiated on IPT
Monthly tracer item stockout rate

Abbreviation: ANC, antenatal care.

As a means of addressing possible non‐parallel trends between enrolled and non‐enrolled facilities we match facilities on pre‐baseline DHIS variables under the assumption that facilities with similar trends in these observables have time‐varying unobservables which evolve similarly.^21^ If we believe these pre‐treatment measures of activity are correlated with factors that may impact facility quality measures then matching improves comparability of facilities. Specifically, returning to potential concerns around differences in facility effort or capacity, matching on these activity factors should increase comparability between enrolled and non‐enrolled facilities, reducing reliance on the assumption that the effect of these unobservables is constant over time.

Because matching cannot distinguish systematic trend differences from short‐term fluctuations due to random shocks, we aggregate monthly DHIS variables to quarterly averages (Linder and McConnell, 2018). Supporting Information S1: Appendix M presents graphs of the trends in the pre‐treatment DHIS variables for both enrolled and non‐enrolled facilities. Although enrolled facilities perform a slightly larger number of services for each of the activities listed, the trends in activity are largely identical.

Given the impracticality of matching on numerous covariates, we match on propensity scores (PS) calculated from these activity measures (Rosenbaum & Rubin, 1983). We estimate PS's using both levels and trends in the DHIS variables (see Supporting Information S1: Appendix N for detail). Balance of the PS and overlap is tested using the standard block method (Becker & Ichino, 2002; Imbens, 2004) and shows good balance between enrolled and non‐enrolled facilities. Further, covariates are shown to be largely balanced between enrolled and non‐enrolled facilities within blocks of the PS. We follow Heckman, Ichimura and Todd (1997a), implementing a kernel propensity‐score matching difference‐in‐difference estimator. Estimation augments the standard DD estimator, whereby instead of controlling for covariates, *X*, in a regression framework, each enrolled facility is matched to the whole sample of non‐enrolled facilities based on weights defined by the propensity score.

The results of the matched DDD analysis, presented in Table 8 suggest that controlling for facilities' previous performance, as measured by DHIS indicators, does not substantially alter the estimated effect of the effect of the QI program.

**TABLE 8:** Kernel propensity score matching difference‐in‐difference.

	Matched on levels				Matched on trends			
	Lowest quality	Low quality	High quality	Highest quality	Lowest quality	Low quality	High quality	Highest quality
2015								
Non‐enrolled	40.20	51.57	59.726	72.02	40.06	51.46	59.963	71.499
Enrolled facilities	41.20	51.85	59.85	71.754	41.20	51.85	59.85	71.754
Difference	1.006 (0.677)	0.281 (0.282)	0.125 (0.267)	−0.266 (0.710)	1.148* (0.674)	0.393 (0.289)	−0.113 (0.282)	0.256 (0.603)
2016								
Non‐enrolled	53.95	57.45	58.57	57.894	53.56	56.49	58.42	58.731
Enrolled facilities	61.12	63.87	71.459	77.267	61.12	63.87	71.459	77.267
Difference	7.163*** (1.881)	6.418*** (1.585)	12.889*** (2.083)	19.373*** (1.389)	7.553*** (1.660)	7.380*** (1.538)	13.04*** (1.695)	18.536*** (1.326)
DD	6.157*** (1.929)	6.137*** (1.656)	13.638*** (2.105)	19.639*** (1.606)	6.406*** (1.721)	6.987*** (1.595)	13.152*** (1.771)	18.28*** (1.388)
Observations	818	956	994	1052	798	942	964	1042

*Note*: Standard errors in parentheses, *p < 0.1, **p < 0.05, ***p < 0.01.

### 6.3 Alternative control group

At the start of 2015/16 FY, every facility was assigned a year for enrollment in the QI program.^22^ If enrollment year decisions were based consistently on beliefs about which facilities were most likely to benefit, this would imply that facilities assigned for enrollment in the subsequent year (2016/17) would most resemble facilities enrolled during our period of analysis in relevant unobservables. Therefore, these facilities should constitute a more valid control group than all non‐enrolled facilities. If this were the case, and our previous analyses had not adequately captured the aspects of the unobservables which impact enrollment and QS, we would expect estimates of the effect of the QI program with this more targeted control group to be smaller than our previous estimates. Unlike the other specification tests, this test utilizes policy‐makers own prioritization criteria in order to maximize the comparability of enrolled and non‐enrolled facilities. We rerun equation (2) with this targeted control group.

As can be seen from Table 9, although the estimated effects of the QI program using the restricted control group are marginally smaller compared to when estimated using the full sample, this difference is minor. This suggests facilities selected for prioritization are not significantly different than those earmarked for later enrollment. This either allays concerns that prioritized facilities had some unobserved characteristics related to both enrollment and changes in QS, or that if these unobservables are present, such as differences in facility capacity, they do not have a significant impact on facility ICRMP QS.

**TABLE 9:** Multiplicative interaction model with restricted sample.

	FE (full sample)	FE (restricted sample)
QI prog lowest baseline quality (γ)	6.064*** (1.312)	5.431*** (1.501)
QI prog low baseline quality relative to *γ*	0.651 (1.722)	0.328 (2.034)
QI prog high baseline quality relative to *γ*	9.671*** (1.747)	8.615*** (2.060)
QI prog highest baseline quality relative to *γ*	14.74*** (1.769)	13.68*** (2.077)
Year lowest baseline quality	13.57*** (0.734)	14.18*** (1.036)
Year low baseline quality	−8.378*** (1.044)	−8.075*** (1.507)
Year high baseline quality	−17.99*** (1.109)	−16.974*** (1.556)
Year highest baseline quality	−29.10*** (1.202)	−28.010*** (1.625)
R^2	0.403	0.413
F	151.5	117.5
Observations	4727	3458

*Note*: *R‐squared reported is with‐in value.

### 6.4 Select ICRMP component scores

Tables 4 and 5 revealed large secular trends among control facilities. This may raise concerns about the impact of random variation in the measurement of ICRMP QS. As noted, the aggregate ICRMP quality score is made up of approximately 150 indicators across 10 components.

We test whether our results change by using a sub‐group of ICRMP components which have the lowest over‐time variation in control group component‐specific QS. For each of the 10 components we, as before, split facilities into quartiles based on aggregate ICRMP quality score, then calculate the component‐specific quality score change in control facilities. For each quartile‐component group we then calculate the percentage change in control group component‐specific quality score (40 total scores). Within each component we aggregate the relative control facility change for the quartiles to identify the components with the highest and lowest relative changes in control facility component‐specific QS. A similar procedure is used to identify the components with the highest and lower absolute changes in control group scores.^23^ The five components with the lowest aggregate relative and absolute variation in QS among control facility are components 1 (Administration), 2 (Integrated Clinical Services Management), 3 (Medicines, Supplies and Laboratory Services), 7 (Health Information Management) and 8 (Communication). We create an amended aggregate ICRMP quality score using only these five components. Table 10 presents the output of re‐running the fixed effects stratified regressions, analogous to Table 4 on this augmented aggregate quality outcome.

**TABLE 10:** FE stratified regressions using select component scores.

	All facilities	Lowest baseline quality	Low baseline quality	High baseline quality	Highest baseline quality
QI program	9.724*** (0.719)	6.956*** (1.546)	7.031*** (1.242)	15.81*** (1.239)	18.52*** (1.218)
Year	2.203*** (0.536)	14.97*** (0.833)	5.908*** (0.853)	−3.860*** (0.924)	−13.60*** (0.968)
R^2	0.176	0.511	0.305	0.250	0.330
F	163.0	155.0	67.40	60.84	63.90
Observations	4727	1178	1181	1181	1187

As can be seen, the results remain relatively unchanged using the quality score components with the most stable control groups across all baseline quality quartiles.

Further, we examine variation at the indicator‐level. Every indicators can take the value 0 (not achieved), 0.5 (partially achieved) or 1 (achieved). We found that for approximately three quarters (111/151) indicators >50% of facilities saw their score remain constant between 2015/16 and 2016/17, while for one quarter (40/151) over >50% of facilities saw their score change. This suggests there is a degree of state dependence and stability at the indicator level. It may, therefore, be the aggregation of small differences over the 151 elements which is contributing to the large changes in scores observed. This reassures us that although ICRMP QS may exhibit a high degree of variation, this variation likely reflects the accumulation of small legitimate quality changes.

Finally, we identify that 19 out of the 20 indicators with the highest proportion of facilities in the highest baseline quality quartile seeing a decline are ones with indicator‐specific checklists. These indicators have individualized checklists which are aggregated to identify whether the indicator was achieved (>90% indicator‐specific checklist), partially achieved (40%–89% indicator‐specific checklist) or not achieved (<40% indicator‐specific checklist).^24^ This is despite indicators with indicator‐specific checklists constituting less than 30% of total indicators. The fact the indicators exhibiting the highest degree of deterioration among highest baseline quality facilities are the indicators, by virtue of their format, with higher sensitivity to changes in score, further suggests the secular trends observed reflect true changes in quality.

## 7 DISCUSSION

### 7.1 Main findings

The examination of heterogeneous treatment effects are particularly important in circumstances where policy objectives value reducing inequality, and therefore weight is given to the distribution of an outcome. In addition to average low quality, the second stylized fact about health care in LMICs is the high levels of quality variation. There is, therefore, increasing recognition of the importance of addressing these large variations in the quality of health care provision and improving equity in access to high‐quality health care. Understanding distributional treatment effects of QI programmes is vital to guide the design, implementation and adjustment of programmes to ensure they contribute toward improving equitable access to high‐quality health care. While a common objective of QI programmes is to reduce variation in the quality of health care, the literature on evaluations exploring effect heterogeneity and the consequences for variation in the quality of health care is currently limited. This is more surprising given the growing recognition that a potential unintended consequence of programmes can be to exacerbate pre‐existing disparities in health care quality, with the design of a number of QI programmes including features intended to reduce this risk (Eijkenaar, 2013).

This paper explores heterogeneous effects of a QI program in SA, attempting to illuminate the distributional consequences for health care quality. First, we employ a DDD design providing insight into the existence and direction of heterogeneous effects. We then estimate the full counterfactual distribution of the enrolled facilities across baseline quality levels using the CC framework, allowing for a full assessment of effect heterogeneity. The paper fits into the growing literature estimating quantile treatment effects (Bitler et al., 2006, 2008; Callaway et al., 2018; Dammert, 2009; Powell, 2020). Key advantages of the CC model are the provision of more information on the distributional effects of the QI program as well as removing the unrealistic assumption of additivity. If the distribution of effort or facility unobserved characteristics are different between enrolled and non‐enrolled groups, and these unobservable facility characteristics are related to responsiveness to the QI program, the assumptions of the CC model enables a more accurate reflection of how QS are determined. However, this benefit is achieved at the expense of being able to identify the relevant units treatment effects relate to. The scale‐independence of CC is not a significant benefit in our case due to the similar distribution of pre‐treatment QS between enrolled and non‐enrolled facilities reduces its importance in our particular case (Meyer, 1995).

Despite the differences in the interpretation of the treatment effect heterogeneity identified by the DDD and CC methods, the results point toward similar conclusions. All facilities, regardless of pre‐treatment quality score, benefit from the QI program. However, the QI program disproportionately benefits facilities with higher baseline quality. A key objective of the ICRMP is to increase the quality of health care provision in SA across all PHC facilities to a set quality threshold. Our analysis suggest that the program will promote this objective, as well as improving average quality. However, this is occurring at the expense of increased variation in the quality of care. Consequently, the program may—at least in the short term—exacerbate pre‐existing variations in health care quality and inequalities in the provision of quality health care.

As highlighted, there is a general convergence in the QS among facilities across baseline score quartiles for unenrolled facilities (Tables 4, 5, and 8). For facilities with higher baseline QS, the QI program had a protective effect against observed quality score reductions observed among the non‐enrolled facilities, while for facilities of lower baseline quality, the QI program only marginally added to the over‐time improvements of their unenrolled peers. This suggests the QI program may be off‐setting other factors that are causing variation in quality over time for facilities of different baseline QS. While this pattern of quality changes over time among low and high baseline performing facilities resembles a regression to the mean (RTM) effect, unlike traditional RTM, this does not impact the internal validity of our results. The problems of RTM in DD settings are well known (Chabe‐Ferret, 2015; Daw & Hatfield, 2018a, 2018b; Daw & Hatfield, 2018a, 2018a, 2018b, 2018b, 2018b, 2018b; Ryan, 2018). These studies show problems are caused by pre‐treatment outcome levels being correlated with treatment assignment. This is a potentially common issue as health policies are frequently targeted and the standard assumption is that DD can be applied even in circumstances where there are baseline outcome differences between treated and control groups. In our case, the QI program enrollment is distributed across facilities of all baseline quality levels and the baseline QS are very similar between enrolled and unenrolled facilities. In the absence of the QI program, any RTM would cause enrolled and unenrolled facilities to regress back to the same common level. Therefore the treatment effects estimated for each quartile/quantile approximates the true treatment effects of the QI program, even in the presence of differential dynamic trends in quality across facilities. Therefore, in the short term at least, the QI program actively worked against other phenomena which would have reduced variation in the quality of health care. The program traded‐off reduced variation in the quality of care offered by facilities across SA with an improvement in the aggregate score compared to the counterfactual situation where the program was not implemented.

This study, therefore, also reaffirms the importance of heterogeneity analysis generally, in enabling drawing more detailed and nuanced policy implications from the implementation of programmes, but it also allowing for a better understanding of the data. Examination of the aggregate impact of the QI program suggests unenrolled facilities see minimal change in their QS. However, this impression of stability among QS of unenrolled facilities stems from diverging trends among sub‐groups which cancel each other out on aggregate. This is a particularly subtle form of masked effect heterogeneity which we refer to as “aggregation obscured trends.” Therefore, undertaking such heterogeneity analysis provides not only information on distributional effects, but also greater insights into the mechanisms through which the treatment effects are materializing.

### 7.2 The relationship between equity in health facility quality and population health equity

Alongside population health maximization, achieving equity in population health outcomes is often a key objective of health systems. In this sense, it is important to differentiate between equality/equity in health facility quality and equity in population health. It is clear that equality in health facility quality does not necessarily lead to equity in population health. It may be beneficial from a health maximization perspective, or even from the perspective of reducing population health disparities to have facilities provide different quality of care. However, within levels of care, deliberate systematic variation in the quality of health facilities is generally not proposed as a solution to address population health inequities. There are two reasons why, holding the level of care/facility and condition/intervention constant, equality in the quality of health facilities is pursued; an ethical reason and a pragmatic reason.

It is well‐established that significant quality deficits and variation exist across health care providers (Das & Gertler, 2007; Haemmerli et al., 2021; Kruk et al., 2017) and even within providers for different patient sub‐populations (Fink et al., 2019). The Lancet Global Health commission argued that a high quality health system should exhibit an “absence of disparities in the quality of health services between individuals and groups” (Kruk et al., 2018a, 2018b). This implies health care quality should be supplied without geographic disparities. From an ethical perspective, distributing health care quality unequally—even to attempt to offset pre‐existing inequities in population health—voids the principle of equal access to high‐quality health care and contradicts the principle of horizontal equity, ensuring those with equal needs receive equal treatment (Wagstaff et al., 1991). Additionally, a policy that aims to induce systematic variation in facility quality targets the geographic‐level and therefore may, in some instances, worsen individual inequities in health outcomes, as health facilities serve diverse populations facing different levels of disadvantage. From a more pragmatic perspective, ensuring high‐quality care for all is an important factor in ensuring collective buy‐in into health systems from all sections of the population. Assuming a progressive taxation system, richer (and healthier) individuals contribute more toward financing public health care. Therefore, if these individuals systematically received lower quality health care they may ask legitimate questions about why they are financing a deliberately discriminatory health system.

This study does not deal with different levels of care whereby patients may be referred to a higher level or different levels offer different services. The facilities in this study are all PHC facilities offering a similar set of services. Therefore, a patient within the catchment of PHC facility *i* is entitled to the same level of quality as a patient living near PHC facility *j*.

Therefore, although it may be possible to improve population health equity through the deliberate systematic variation in health facility quality, and equity may play a significant role in forming certain supply‐side health policies, deliberately imposing geographical inequalities in the quality of locally accessible health care is almost never pursued. Addressing inequities in population health outcomes is almost always addressed through policy levers targeting the social determinants of health, or ensuring any inequities in access to and utilisation of health care is addressed.

For this reason, equal quality of health facilities may be viewed as equitable, and the “equitable distribution” of health care quality within services is usually viewed as an equal distribution. Consequently, despite the two objectives not always being perfectly aligned, health systems most commonly simultaneously pursue the objectives of equality in the quality of health facilities and equity in population health outcomes. While our study is principally concerned with the former we identify a positive correlation between SES and facility baseline quality.^25^ Therefore, as the QI program acted to increase variation in facility quality compared to the counterfactual scenario where it wasn’t implemented, it may have acted to worsen population health equity.”

### 7.3 Mechanisms behind heterogeneous treatment effects

The focus of this paper is to examine possible distributional effects of the ICRMP QI program. As we do not explicitly examine mechanisms, partially due to data limitations, we are cautious in our interpretation around the mediating causes for the differences in the observed effect of the program. However, understanding the mechanisms behind the observed heterogeneity has obvious policy importance. Previous studies of QI programmes, notably PBF schemes, have hypothesized factors that may determine both past and present health care quality and the effectiveness and responsiveness of health facilities to improvement programmes. Disentangling the effect of various factors on the impact of QI programmes is difficult due incentive structures often interacting with facility characteristics, and various determinants of past quality likely impacting facilities ability to respond to programmes.^26^ This introduces challenges in separating the impacts of the various determinants of past performance have on responsiveness to QI programmes (Markovic & Ryan, 2017). In our setting, idiosyncratic program features—specifically the quality indicators measured and the additional resources—may provide insight into factors impacting both past quality performance and QI program responsiveness.^27^ Unlike PBF programmes, where theory predicts baseline low performers face higher marginal costs in responding to programmes, there is limited rationale for differential responses to the ICRMP QI program (Mullen et al., 2010). Compared to target‐based financial reward QI schemes such as results based financing/P4P where incentive structures of these schemes can interact with provider characteristics. Economic theory predicts differential provider responses due to differences in marginal costs. If costs are related to past performance, very low performers should face higher marginal costs, while very high performers may face higher marginal costs as this usually increases in quality. In our context, based on the indicators examined, there is no strong rationale for substantial variation in the marginal cost of quality improvement across facilities with differential past performance. Further, Dowd et al. (2012), that there is no “give up” effect of being a low baseline performing health care provider in the face of absolute quality targets, even if reaching performance targets is unrealistic. If we observed “progressive” treatment effects of the ICRMP QI program, this may have hinted toward previous capacity constraints (e.g., financial resources) faced by low baseline quality facilities being responsible for past poor performance, as the program relaxing this constraint would allow previous low performers to improve quality. However, because unenrolled poor performing facilities also saw their ICRMP QS increase, this suggests previously poor quality facilities were not performing at their technical efficiency frontier with respect to quality, or were at least able to improve quality without additional financial resources. Therefore, while we do not directly examine between facility variation in pre‐treatment quality, our results enable us to speculate on factors explaining observed pre‐existing facility quality variation, and which may be important in modifying facility’s QI program responsiveness. While our analysis is largely restricted to attempts to identify the impact of the QI program for facilities and how this impact may vary across facilities of different pre‐treatment quality performance, this illustrates how examining effects beyond the mean may provide introductory insights regarding important effect mediators. However, in order to formally assert such claims about mechanisms, a full mediation analysis would be required.

### 7.4 Limitations

Our identification strategy is susceptible if there is selection into QI enrollment based on underlying time trends in facility quality this would bias estimates of the ATT. We have attempted to control for the various sources which may contribute to such differential trends with the DDD and CC identification strategies ruling out differences in time‐invariant factors contributing to differential trends. Additionally our sensitivity checks ensure enrolled and unenrolled facilities are as comparable as possible on a range of pre‐treatment observables and related unobservables. Although we try to control for key time‐varying facility‐level variables that may determine QI enrollment and have an independent influence on QS, it is not only contemporaneous differences in changes across time‐varying inputs which may be problematic. If facilities viewed as improving had received increases in their inputs pre‐treatment, the full effect of these inputs may occur over a period of time. Therefore, pre‐treatment changes in facility inputs or circumstances with a lagged effect may also impact QS. Our matching sensitivity analysis should account for this by comparing facilities with similar pre‐treatment facility activity accounting for changes in pre‐treatment observables with a lagged effect and correlated unobservables.

We only have two‐periods and two‐groups in which to assess the impact of the QI program. In such cases, inference is dependent on the structure of uncertainty (Lechner, 2011). Despite exploring the constituent sub‐components of ICRMP QS, the lack of additional pre‐treatment scores restricts our ability to fully understand the natural trend and variation in its measurement. This highlights the issue of impact assessment using recently introduced indicators and measures, limiting the ability to explore its properties. Additionally, this restricts inference to assessment of short‐term effects of the QI program. On the other hand, analysis on only two periods ensures that any bias imparted from a violation of the parallel trends assumption is bounded by the single year maximum difference in the trends, as opposed to a cumulative function of trend differentials, and therefore increases our confidence that the true pattern of heterogeneity is close to that observed.

Finally, there is ongoing debate regarding measures of quality of health care (Akachi & Kruk, 2017). The quality measures used in the ICRMP are largely restricted to structural and process measures of quality (Donabedian, 1966; McIntyre & Ataguba, 2018). While there is evidence that accreditation type QI programmes may improve such structural and process measures, there is less evidence that this translates into improved health outcomes. However the focus of this study, emphasizing the importance of measuring distributional effects and examining how this may be done, is equally applicable to all QI program indicators types.

Future studies seeking to examine differential provider responses to QI initiatives would benefit from including quality measures along all quality dimensions—structural, process and outcome; better data on the long run dynamics of all quality dimensions of interest (i.e., trends, stationarity etc.) and continued measurement post‐initiative to identify long‐run impacts of QI programmes and the sustainability of any improvements achieved.

## 8 CONCLUSION

Inequality in access to high‐quality health care is a prominent issue in many LMICs. Efforts to promote higher quality health care, including QI programmes, are being increasingly implemented. Key objectives of QI programmes often include promoting minimum quality standards or reducing variation in the quality of health care provided, with strong efforts dedicated in program design to promote such objectives. Even when equity is not an explicit objective, the equity consequences of a policy should be reported. Larger effects in reducing negative health outcomes attributable to low‐quality care are likely to be observed when programmes disproportionately impact suppliers at the bottom end of the quality distribution. We therefore contend that future evaluations of QI programmes should not limit themselves to the examination of mean impact. The case for focusing solely on mean impacts is that undesirable distributional aspects of policies are either unimportant or can be offset by transfers (Heckman et al., 1997a, 1997b). Neither of these are true in the case of QI programmes in health care. Consequently, evaluations should be undertaken with equality objectives in mind and programmes constructed to target the sources of these inequalities.

## CONFLICT OF INTEREST STATEMENT

No conflicts of interest are present to declare.

## Supporting information

Supporting Information S1

## Data Availability

The data that support the findings of this study are available from South Africa National Department of Health. Restrictions apply to the availability of these data, which were used under license for this study. Access to data can be sought via the permission of the National Department of Health.

## ENDNOTES

^1^
They distinguish this from clinical supervision which have more of a medical education agenda and are focused specifically on clinical practice. Managerial supervision is more wide‐ranging and an important part of the link between peripheral health care providers and district‐level policy makers. It is often part of regular district management procedures and examines administrative and managerial activities as well as clinical procedures.

^2^
It should be noted that heterogeneous treatment effects is not a singular concept. Here we restrict exploration to when treatments interact with pre‐treatment variables, sometimes referred to as treatment‐covariate interactions. Subsequently, we narrow our discussion to assessment of heterogeneity across pre‐defined, discrete sub‐groups. However, another source of heterogeneity may be apriori partial knowledge of potential gains, resulting in possible correlation between the treatment effect size and the probability of receiving treatment (Heckman et al., 2006; Manski, 1990). The presence of the latter leads to questions on the validity of standard treatment effect estimates. See Supporting Information S1 for a more formal distinction between sources and types of treatment effect heterogeneity.

^3^
Also known as pay for performance (P4P), Results based financing (RBF).

^4^
In any given study there are numerous sub‐populations across which samples can be split to test for treatment effects when there is a belief these may be heterogeneous. This is one of several ways issues associated with multiple hypothesis testing can arise increasing the possibility of type I error (List et al., 2019).

^5^
There is general missing data in addition to Western Cape not participating during 2015/16 FY and therefore not having checklist information.

^6^
This was done using QGIS.

^7^
Supporting Information S1: Appendix D shows the geographical distribution of facilities by ICRMP enrollment and baseline quality strata across Districts in SA. The table illustrates that facilities of different baseline quality are well distributed geographically with limited evidence of spatial clustering. Further, ICRMP enrollment is well distributed across baseline quality strata within districts. These factors are important for two reasons. If facility ICRMP enrollment were spatially clustered by Districts we may be concerned that these Districts systematically vary in not only their levels of facility quality but also their ability to impact changes in facility quality in ways that may be unrelated to ICRMP enrollment. Second, if Districts were enrolling facilities in the ICRMP by baseline stratum, concerns would arise that authorities may systematically focus on these facilities in other ways. Given there appears to be limited evidence of either issue taking place, suggests geographical controls may not be fundamental to any identification strategy.

^8^
In most models with interaction terms it is important to include main effects for interpretation purposes but when estimating FE models this is not possible.

^9^
Specifically, the linear interaction effect assumes the effect of treatments varies at a constant rate across levels of the moderator examined. In this case this would imply ∂Y∂QI=α+γBaseQulf.

^10^
Hainmueller et al. (2019) also highlight the requirement of common support over moderators with such interactive models. However, common support issues are less pressing with discrete moderator variables and reassuringly Table 4 showed strong common support of the treatment across the moderator strata.

^11^
Time‐invariant covariates whose effects vary over time must be included in the model as FE estimation only controls for time‐invariant variables with time‐invariant effects (Allison, 2009).

^12^
Unlike an LDV specification, our dependent variable remains the within facility change in quality score.

^13^
Because of how the counterfactuals are identified with the CC, it is not possible, without strong assumptions, to clearly identify units or groups to which the treatment effect relates and reference can only be made to points in the distribution. In order for the QTT to reflect the treatment effect for a particular unit an implausibly strong assumption of rank preservation needs to hold. This would require that each facility maintains its rank across the (potential) outcome distributions.

^14^
We use the cic STATA command (Melly & Santangelo, 2015) to obtain conditional CC estimates.

^15^
A benefit of the stratified approach is the ease of interpretation of coefficients which are given in absolute terms rather than relative to the baseline strata (lowest baseline quality).

^16^
Namely the average monthly nurse working days and average monthly patient headcounts at facility.

^17^
Note the distribution is that of the pre‐treatment quality for the enrolled facilities, therefore, the quantiles along which the treatment effects (QTTs) are calculated and presented are with respect to this CDF.

^18^
Further, if there is differential trends in pre‐treatment quality and this is caused by facility managerial quality or some other facility unobserved effects, these are now no longer controlled for.

^19^
This also prevents us from pursuing a modeling strategy which simultaneously controls for unobserved fixed effects and past outcomes. Although this saves us having to address the known challenges with such models (Nickell, 1981).

^20^
It is worth clarifying that although the DDD specifications captured a measure of the baseline quality measure in the RHS (namely a categorical variable indicating pre‐treatment quartile) this only allowed the parameters of the treatment and time trend to vary across these groups and remains distinct from the LDV specification.

^21^
This is a slight adaptation of the popular approach which matches on pre‐treatment outcomes then applies DD which is used to address non‐parallel trends (Blundell & Dias, 2009).

^22^
Originally the plan was for all facilities to be enrolled within 3 years. Therefore, approximately 1000 facilities were assigned for enrollment per year between 2015/16–2017/18.

^23^
For both the aggregate relative and aggregate absolute control facility score change we use absolute values, with negative percentages and integers treated as positives.

^24^
There is some variation with some indicators requiring either 80% or 100% to judge the indicator as being achieved.

^25^
Result available upon request.

^26^
Due to concerns around factors influencing past performance also influencing PBF responsiveness, many PBF‐style programmes implement prior facility readiness assessments and structural and process quality equalizing investments.

^27^
Unlike previous studies, observed heterogeneity should not be unduly influenced by health facility's individual capacities, notably financial resources. This is due to a the indicators included in the ICRMP checklist which relate primarily to structural and process measures of health care quality which lie largely within the influence of facilities own actions. Additionally, for the select indicators which do require external assistance such as direct financing, this was provided as part of the QI program. Differences in the ability of facilities to attain targets is what causes programmes to introduce differential incentives and targets and further complicates learning about the determinants of part performance and responsiveness to QI programmes.
