# Supplementary material for: Do health care quality improvement policies work for all? Distributional effects by baseline quality in South Africa
Source: Health Econ. 2024 Oct 3;34(1):175–99. doi: 10.1002/hec.4899 (PMC11631827; doi:10.1002/hec.4899)
Supplement: Supplementary file 1 — Supporting Information S1 [file HEC-34-175-s001.docx]

**Main Manuscript Appendix**

**Appendix A**

An ‘Ideal Clinic’ is defined as a clinic with:

- Good infrastructure (i.e. physical condition and spaces, essential equipment, and information and communication tools)
- Adequate staff
- Adequate medicines and supplies
- Good administrative processes
- Adequate bulk supplies; such a clinic uses applicable clinical policies, protocols and guidelines, as well as partner and stakeholder support, to ensure the provision of quality health services to the community.


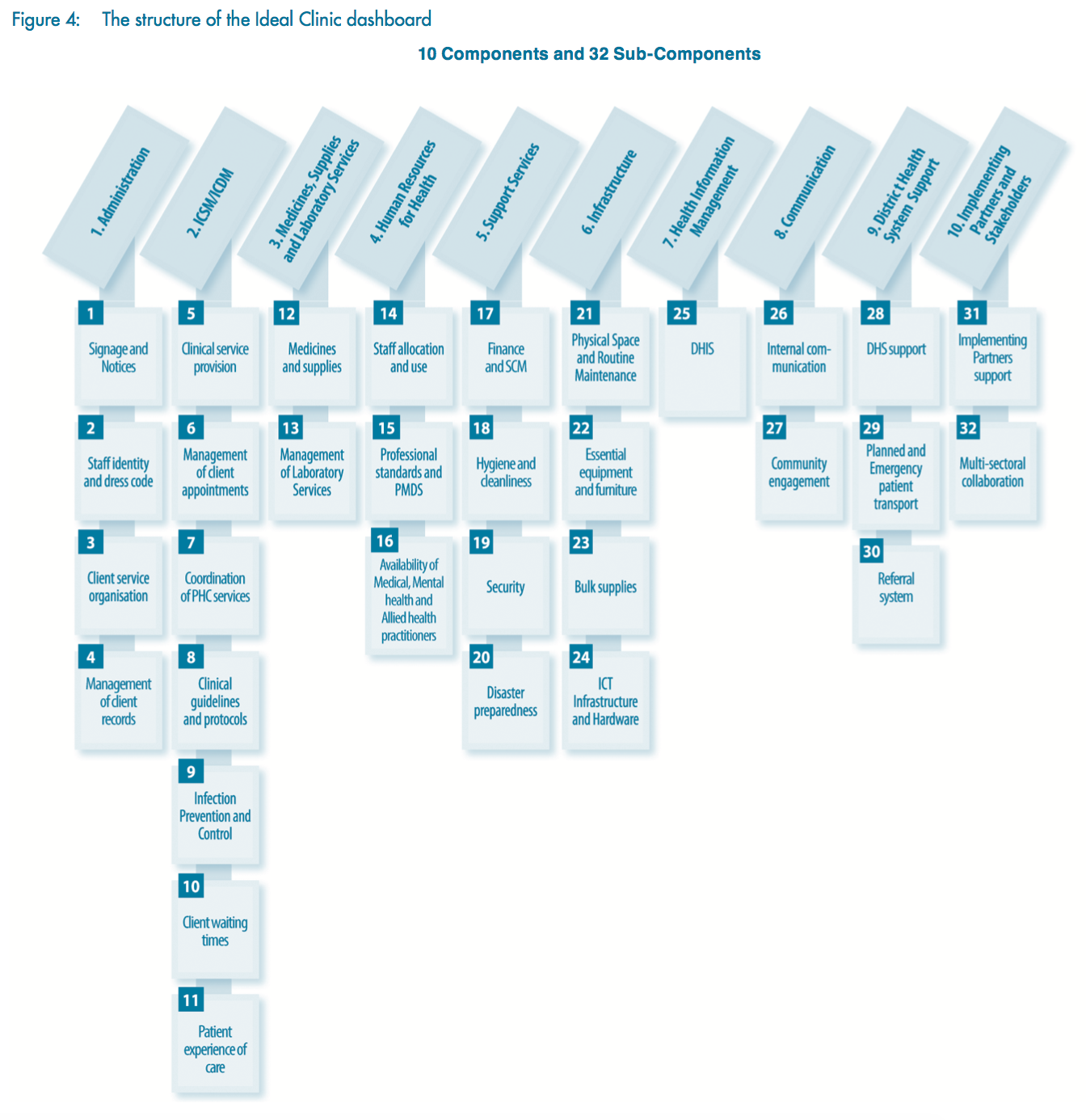


See end of document for full indicator list for ICRMP (**Appendix A Cont.**)

**Appendix B**

Figures 1(a) & 1(b): Distribution of ICRMP quality scores across baseline quality strata in 2015 and 2016


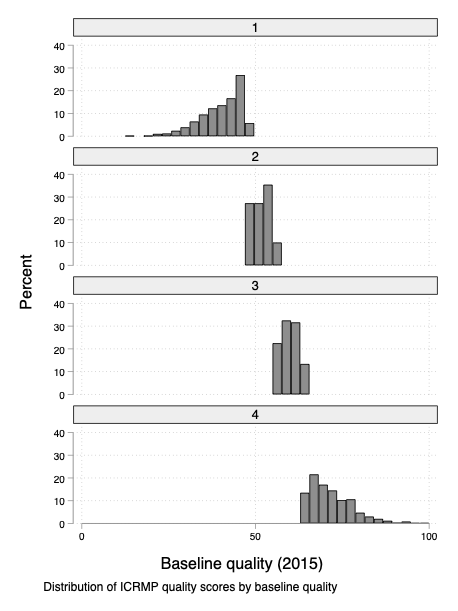


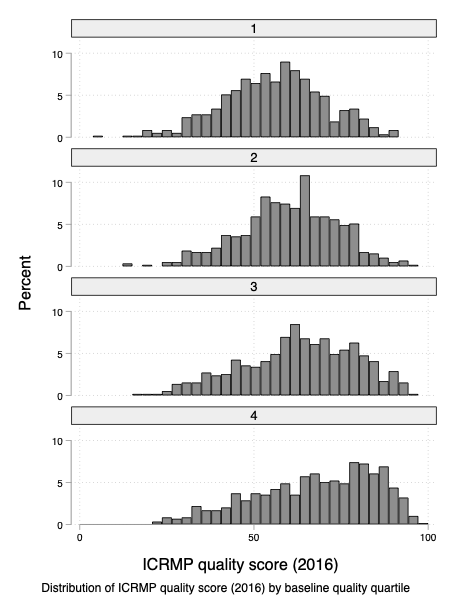


**Appendix C**

Appendix Tables 2(a)-3(b) present ANOVA examining whether the average ICRMP quality scores are significantly different among facilities of different sizes as measured by monthly patient headcounts and the monthly number of nurse working days. Both variables pass Barlett’s test for equal variances.

| Appendix Table 2(a): Summary for facility size (patient headcount (2015)) and baseline quality score | | | |
| --- | --- | --- | --- |
| Quartile of average monthly patient head count across year (2015) | Mean ICRMP Quality Score | Standard deviation | Frequency |
| 1 | 54.28 | 12.55 | 498 |
| 2 | 55.55 | 12.76 | 559 |
| 3 | 55.33 | 12.29 | 645 |
| 4 | 57.85 | 12.15 | 670 |
| Total | 55.87 | 12.48 | 2,372 |

| Appendix Table 2(b): ANOVA test for facility size (patient headcount (2015)) and baseline quality score | | | | | |
| --- | --- | --- | --- | --- | --- |
|  | Sum of Squares | degrees of freedom | Mean square | F-statistic | significance |
| Between group | 4127 | 3 | 1376 | 8.92 | 0.00 |
| Within group | 365216 | 2368 | 154 |  |  |
| Total | 369,343 | 2371 | 156 |  |  |

| Appendix Table 3(a): Summary for facility size (nurse working days (2015)) and baseline quality score | | | |
| --- | --- | --- | --- |
| Quartile of average monthly nurse working days across year (2015) | Mean ICRMP Quality Score | Standard deviation | Frequency |
| 1 | 53.97 | 12.49 | 458 |
| 2 | 55.45 | 12.72 | 563 |
| 3 | 55.9 | 12.25 | 668 |
| 4 | 57.49 | 12.33 | 680 |
| Total | 55.87 | 12.48 | 2,369 |

| Appendix Table 3(b): ANOVA test for facility size (nurse working days (2015)) and baseline quality score | | | | | |
| --- | --- | --- | --- | --- | --- |
|  | Sum of Squares | degrees of freedom | Mean square | F-statistic | significance |
| Between group | 3533 | 3 | 1178 | 7.62 | 0.00 |
| Within group | 365496 | 2365 | 155 |  |  |
| Total | 369030 | 2368 | 156 |  |  |

As can be seen the ANOVA results suggest facilities with higher monthly patient visits and nurse working days have statistically significantly higher baseline (2015) quality scores. Figures 2(a) and 2(b) reveal that much of the difference in baseline quality between facilities of different sizes may be driven by a few much larger facilities, measured by patient headcount and nurse working days, which have higher quality scores. For instance, the facility at the 95^th^ percentile for patient headcount saw on average 6,957 patients per month in 2015, while the 99^th^ and 100^th^ percentiles see 15,651 and 41,366 patients per month. Average nurse working days is similarly right-skewed across facilities.

Figure 2(a) & 2(b): Facility size and baseline quality scores


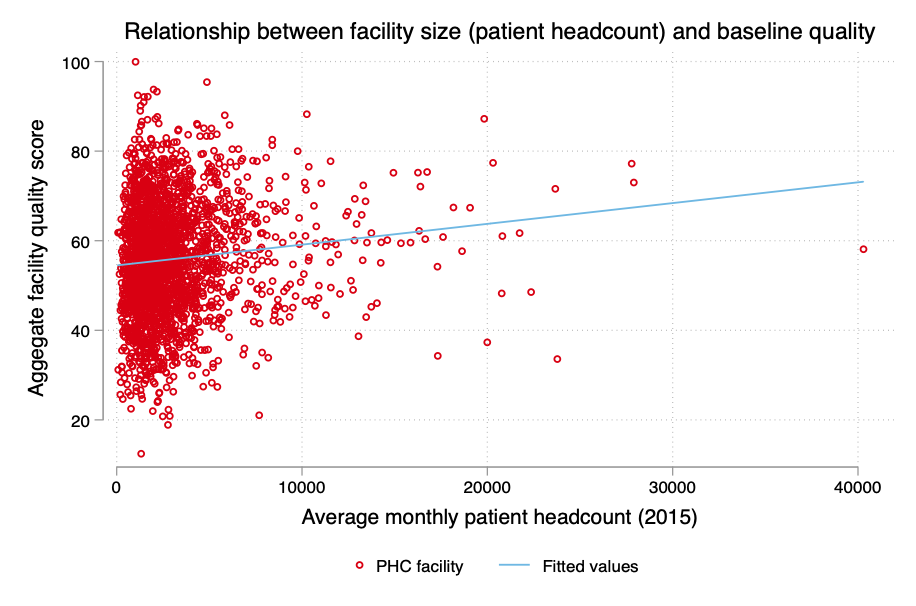


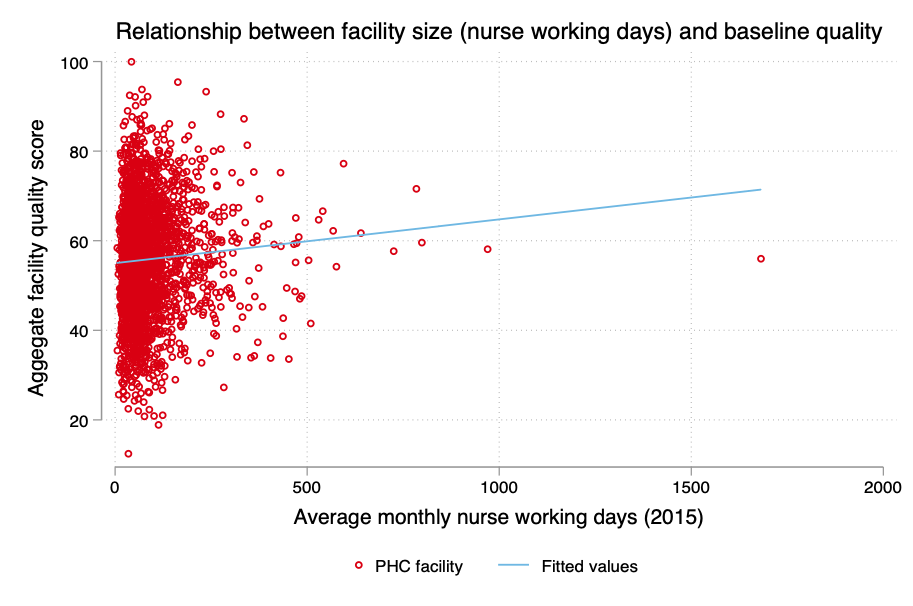


**Appendix D**

| **Appendix Table 2: Facility baseline quality status and ICRMP enrolment by District** | | | | | | | | | | |
| --- | --- | --- | --- | --- | --- | --- | --- | --- | --- | --- |
|  | **All facilities** | | **Lowest Base Q** | | **Low Base Q** | | **High Base Q** | | **Highest Base Q** | |
| **Districts** | **Non-enrolled** | **Enrolled** | **Non-enrolled** | **Enrolled** | **Non-enrolled** | **Enrolled** | **Non-enrolled** | **Enrolled** | **Non-enrolled** | **Enrolled** |
| A Nzo DM | 5 | 17 | 3 | 5 | 0 | 9 | 2 | 3 | 0 | 0 |
| Amajuba DM | 18 | 8 | 8 | 0 | 5 | 0 | 4 | 4 | 1 | 4 |
| Amathole DM | 12 | 40 | 8 | 7 | 2 | 11 | 1 | 14 | 1 | 8 |
| Bojanala Platinum DM | 68 | 40 | 15 | 3 | 16 | 10 | 19 | 11 | 18 | 16 |
| Buffalo City MM | 1 | 26 | 0 | 7 | 1 | 11 | 0 | 7 | 0 | 1 |
| C Hani DM | 24 | 31 | 3 | 5 | 6 | 8 | 6 | 11 | 9 | 7 |
| Capricorn DM | 63 | 37 | 39 | 17 | 15 | 14 | 8 | 4 | 1 | 2 |
| Dr K Kaunda DM | 27 | 12 | 5 | 1 | 16 | 4 | 5 | 7 | 1 | 0 |
| Ehlanzeni DM | 61 | 38 | 15 | 14 | 16 | 16 | 20 | 5 | 10 | 3 |
| Ekurhuleni MM | 39 | 49 | 0 | 0 | 5 | 2 | 13 | 11 | 21 | 36 |
| Fezile Dabi DM | 18 | 20 | 5 | 1 | 5 | 6 | 7 | 8 | 1 | 5 |
| Frances Baard DM | 11 | 12 | 2 | 1 | 4 | 7 | 2 | 2 | 3 | 2 |
| G Sibande DM | 43 | 21 | 19 | 5 | 10 | 5 | 8 | 9 | 6 | 2 |
| Harry Gwala DM | 28 | 1 | 4 | 0 | 5 | 0 | 4 | 1 | 15 | 0 |
| J T Gaetsewe DM | 27 | 13 | 21 | 2 | 3 | 5 | 3 | 5 | 0 | 1 |
| Joe Gqabi DM | 1 | 17 | 0 | 1 | 0 | 5 | 1 | 4 | 0 | 7 |
| Johannesburg MM | 48 | 54 | 18 | 17 | 16 | 15 | 10 | 14 | 4 | 8 |
| King Cetshwayo DM | 39 | 19 | 10 | 0 | 10 | 0 | 8 | 6 | 11 | 13 |
| Lejweleputswa DM | 2 | 20 | 1 | 7 | 1 | 7 | 0 | 4 | 0 | 2 |
| Mangaung MM | 2 | 21 | 2 | 2 | 0 | 9 | 0 | 7 | 0 | 3 |
| Mopani DM | 53 | 34 | 13 | 9 | 12 | 11 | 13 | 5 | 15 | 9 |
| N Mandela Bay MM | 4 | 17 | 1 | 8 | 1 | 4 | 1 | 3 | 1 | 2 |
| Namakwa DM | 22 | 11 | 18 | 1 | 4 | 6 | 0 | 2 | 0 | 2 |
| Ngaka Modiri Molema DM | 52 | 28 | 36 | 14 | 14 | 13 | 2 | 0 | 0 | 1 |
| Nkangala DM | 60 | 26 | 18 | 2 | 12 | 9 | 13 | 9 | 17 | 6 |
| O Tambo DM | 4 | 41 | 2 | 5 | 1 | 7 | 0 | 14 | 1 | 15 |
| Pixley ka Seme DM | 21 | 15 | 7 | 3 | 7 | 4 | 6 | 6 | 1 | 2 |
| Ruth Segomotsi Mompati DM | 34 | 20 | 4 | 0 | 4 | 3 | 14 | 6 | 12 | 11 |
| Sarah Baartman DM | 0 | 20 | 0 | 9 | 0 | 3 | 0 | 6 | 0 | 2 |
| Sedibeng DM | 11 | 11 | 3 | 3 | 3 | 1 | 4 | 5 | 1 | 2 |
| Sekhukhune DM | 53 | 29 | 36 | 9 | 11 | 8 | 5 | 9 | 1 | 3 |
| T Mofutsanyana DM | 26 | 30 | 5 | 6 | 10 | 7 | 7 | 7 | 4 | 10 |
| Tshwane MM | 36 | 35 | 3 | 3 | 11 | 5 | 9 | 9 | 13 | 18 |
| Ugu DM | 31 | 22 | 0 | 1 | 9 | 0 | 8 | 6 | 14 | 15 |
| Umkhanyakude DM | 37 | 19 | 2 | 0 | 7 | 1 | 12 | 3 | 16 | 15 |
| Umzinyathi DM | 31 | 3 | 3 | 0 | 10 | 1 | 10 | 0 | 8 | 2 |
| Uthukela DM | 23 | 13 | 1 | 0 | 3 | 0 | 3 | 3 | 16 | 10 |
| Vhembe DM | 71 | 45 | 11 | 3 | 15 | 11 | 28 | 18 | 17 | 13 |
| Waterberg DM | 40 | 9 | 8 | 0 | 8 | 0 | 19 | 1 | 5 | 8 |
| West Rand DM | 19 | 23 | 5 | 6 | 11 | 6 | 3 | 6 | 0 | 5 |
| Xhariep DM | 0 | 9 | 0 | 0 | 0 | 1 | 0 | 3 | 0 | 5 |
| ZF Mgcawu DM | 13 | 8 | 4 | 3 | 4 | 3 | 4 | 1 | 1 | 1 |
| Zululand DM | 40 | 30 | 12 | 1 | 17 | 2 | 2 | 9 | 9 | 18 |
| eThekwini MM | 63 | 16 | 24 | 4 | 10 | 3 | 16 | 7 | 13 | 2 |
| iLembe DM | 20 | 11 | 5 | 1 | 8 | 3 | 4 | 2 | 3 | 5 |
| uMgungundlovu DM | 34 | 17 | 7 | 1 | 6 | 3 | 9 | 2 | 12 | 11 |
| **Total** | **1335** | **1038** | **406** | **187** | **334** | **259** | **313** | **279** | **282** | **313** |

**Appendix E**

| **Appendix Table 1: Distribution of ICRMP enrolment over baseline quality** | | | | |
| --- | --- | --- | --- | --- |
|  | Lowest Baseline Quality | Low Baseline Quality | High Baseline Quality | Highest Baseline Quality |
| Not enrolled in QI | 403 | 334 | 313 | 281 |
| Enrolled in QI | 187 | 257 | 277 | 313 |
| Proportion of QI enrolled | 32% | 43% | 47% | 53% |
| Total | 590 | 591 | 590 | 594 |

**Appendix F**

Baseline quality should be thought of as a component of the facility-level fixed-effects which is interacted with the treatment variable. O’Neil et al. (2016) note that inclusion of a past outcome does not always result in a dynamic model. Specifically, when estimation includes a fixed vector of pre-treatment outcomes, whereas dynamic models define the vector of past outcomes used relative to the current period. This has important implications for the consistency of estimation. Because baseline quality is time-invariant, the main effects are not included in the models. The interaction between QI programme enrolment and baseline quality measures how within facility enrolment in the QI programme effects ICRMP quality scores at different levels of baseline quality. Omission of the main effect of baseline quality is allowed as it is captured in the facility-level fixed effects, $A_{f}$. Giesselmann & Schmidt-Catran (2018) illustrate how interactions in FE regressions when one of the interacted variables has no intra-unit variation, as opposed to both variables having intra-unit variation, allows the identification of within-unit effects across levels of time-constant characteristics, maintaining the within unit effect interpretation. Following Giesselmann & Schmidt-Catran (2018), as ${BaseQul}_{f}$ is time-constant ${BaseQul}_{ft}=\bar{BaseQul}_{f}$ for all $\{f,t\}$ the demeaned interaction, ${BaseQul}_{f}*{QI}_{ft}-\bar{(BaseQul*QI)}_{f}$, collapses to:

$$\left( \bar{BaseQul}_{f}*{QI}_{ft} \right)-\frac{\sum_{t=1}^{T_{i}} \bar{BaseQul}_{f}*{QI}_{ft}}{T_{i}}$$

$$=\left( \bar{BaseQul}_{f}*{QI}_{ft} \right)-\bar{BaseQul}_{f}\frac{\sum_{t=1}^{T_{i}} {QI}_{ft}}{T_{i}}$$

$$=\bar{BaseQul}_{f}({QI}_{ft}-\frac{\sum_{t=1}^{T_{i}} {QI}_{ft}}{T_{i}})$$

$$=\bar{BaseQul}_{f}({QI}_{ft}-\bar{QI}_{f})$$

**Appendix G**

The CC model relies on 4 key assumptions:

A1. In the absence of the QI programme, quality scores (QS), are generated by an unknown function of two random variables; $QS=h(U,T)$. Where $U$ is a vector capturing unobservable facility characteristics and $T$ is time. In this model, therefore, the outcome doesn’t depend on whether a facility is part of the enrolled group $QI$ or not, but $U$ may differ across $QI$. Therefore, for a facility with $U=u$, the outcome, $QS$ will be the same in a given period, $T=t$, whether the facility is part of the enrolled group or not.

A2. $QS=h(U,T)$ is strictly monotone increasing, i.e. $\Delta h\left( U \right)>0$, so higher unobservables result in strictly higher outcomes.

A3. The distribution of unobservable facility characteristics are time invariant within both enrolled and non-enrolled groups, $U\perp T|QI$.

A4. There is support $U|QI=1\subseteq U|QI=0$. From the above, this implies $QS\left| QI=1,t=0\subseteq QS \right|QI=0,t=0$, that the support of the non-enrolled facilities QS is enclosed within the support of the QI enrolled facilities in the pre-treatment period.

**Appendix H**

With Melly & Santangelo (2015) approach including covariates, the production function can be represented as $QS=h(U,T, X)$. Like before, unobservable facility characteristics can differ between enrolled and non-enrolled facilities, but now the time-invariance of the unobservables is $U\perp T|QI,X$.

**Appendix I**

|  |  |  |  |  |  |
| --- | --- | --- | --- | --- | --- |
| **Appendix Table 3: ICRMP Checklist Scores for Enrolled & Non-enrolled** | | | | | |
|  | **Lowest baseline quality facilities** | **Low baseline quality facilities** | **High baseline quality facilities** | **Highest baseline quality facilities** | **All facilities** |
| Average (SD) total quality 2015 non-enrolled | 39.6 (6.3) | 51.6 (2.5) | 59.8 (2.4) | 71.9 (6.6) | 54.1 (12.8) |
| Average (SD) total quality 2015 enrolled | 41.2 (5.2) | 51.8 (2.4) | 59.8 (2.4) | 71.8 (5.8) | 58.1 (11.7) |
| Observations Non-enrolled | 409 | 334 | 313 | 282 | 1,338 |
| Observations Enrolled | 187 | 261 | 282 | 313 | 1,043 |

**Appendix J**

Figure 3(a): CC model without covariates

Figure 3(b): CC model without covariates

**Appendix K**

|  |  |  |  |  |  |  |  |  |
| --- | --- | --- | --- | --- | --- | --- | --- | --- |
| **Appendix Table 4: Changes-in-Changes models** | | | | | | | | |
|  | **Model without covariates** | | | | **Model with covariates** | | | |
| **Quantile** | **Effect** | **Standard Error** | **Uniform Lower Bound** | **Uniform Upper Bound** | **Effect** | **Standard Error** | **Uniform Lower Bound** | **Uniform Upper Bound** |
| 0.01 | 2.4 | 2.72 | -6.82 | 11.62 | 4.76 | 3.05 | -3.36 | 12.87 |
| 0.02 | 3.08 | 2 | -3.72 | 9.89 | 4.37 | 2.10 | -1.21 | 9.96 |
| 0.03 | 5.11 | 2.2 | -2.36 | 12.58 | 6.65 | 1.82 | 1.80 | 11.49 |
| 0.04 | 6.04 | 2.05 | -0.91 | 12.99 | 7.79 | 1.79 | 3.02 | 12.56 |
| 0.05 | 6.01 | 1.93 | -0.56 | 12.57 | 7.54 | 1.80 | 2.75 | 12.32 |
| 0.06 | 6.24 | 1.97 | -0.46 | 12.93 | 6.67 | 1.95 | 1.47 | 11.88 |
| 0.07 | 6.95 | 1.86 | 0.63 | 13.27 | 7.73 | 2.05 | 2.28 | 13.18 |
| 0.08 | 7.21 | 1.68 | 1.51 | 12.91 | 7.72 | 1.87 | 2.73 | 12.71 |
| 0.09 | 7.35 | 1.49 | 2.28 | 12.41 | 7.76 | 1.46 | 3.88 | 11.64 |
| 0.1 | 7.25 | 1.35 | 2.66 | 11.84 | 7.77 | 1.20 | 4.58 | 10.97 |
| 0.11 | 7.41 | 1.31 | 2.98 | 11.84 | 7.65 | 1.04 | 4.89 | 10.42 |
| 0.12 | 7.9 | 1.3 | 3.47 | 12.33 | 7.75 | 0.94 | 5.24 | 10.26 |
| 0.13 | 8.25 | 1.29 | 3.89 | 12.61 | 7.96 | 0.90 | 5.56 | 10.36 |
| 0.14 | 7.78 | 1.38 | 3.1 | 12.47 | 8.20 | 0.88 | 5.85 | 10.55 |
| 0.15 | 8.01 | 1.35 | 3.42 | 12.6 | 8.24 | 0.91 | 5.83 | 10.65 |
| 0.16 | 8.43 | 1.36 | 3.82 | 13.03 | 8.26 | 0.89 | 5.89 | 10.63 |
| 0.17 | 8.79 | 1.35 | 4.2 | 13.39 | 8.42 | 0.93 | 5.95 | 10.89 |
| 0.18 | 9.06 | 1.42 | 4.24 | 13.87 | 8.53 | 1.01 | 5.83 | 11.22 |
| 0.19 | 8.75 | 1.55 | 3.5 | 14 | 8.50 | 1.17 | 5.38 | 11.62 |
| 0.2 | 8.18 | 1.58 | 2.8 | 13.56 | 8.52 | 1.31 | 5.03 | 12.01 |
| 0.21 | 8.46 | 1.45 | 3.52 | 13.39 | 8.71 | 1.29 | 5.27 | 12.14 |
| 0.22 | 8.96 | 1.28 | 4.61 | 13.31 | 8.91 | 1.28 | 5.51 | 12.31 |
| 0.23 | 9.18 | 1.17 | 5.21 | 13.15 | 9.00 | 1.22 | 5.75 | 12.26 |
| 0.24 | 9.06 | 1.09 | 5.34 | 12.78 | 9.05 | 1.18 | 5.92 | 12.18 |
| 0.25 | 8.9 | 1.04 | 5.38 | 12.42 | 9.00 | 1.12 | 6.02 | 11.99 |
| 0.26 | 8.92 | 1.01 | 5.49 | 12.35 | 8.96 | 1.08 | 6.09 | 11.82 |
| 0.27 | 8.68 | 1.02 | 5.22 | 12.13 | 8.84 | 1.06 | 6.01 | 11.67 |
| 0.28 | 8.58 | 1.06 | 4.98 | 12.18 | 8.75 | 1.02 | 6.04 | 11.47 |
| 0.29 | 8.58 | 1.06 | 4.98 | 12.18 | 8.68 | 0.95 | 6.15 | 11.21 |
| 0.3 | 8.77 | 1.08 | 5.12 | 12.42 | 8.70 | 0.88 | 6.35 | 11.05 |
| 0.31 | 8.52 | 1.11 | 4.76 | 12.28 | 8.77 | 0.84 | 6.53 | 11.01 |
| 0.32 | 8.69 | 1.12 | 4.9 | 12.48 | 8.91 | 0.82 | 6.73 | 11.08 |
| 0.33 | 9.38 | 1.1 | 5.65 | 13.11 | 9.02 | 0.80 | 6.89 | 11.14 |
| 0.34 | 9.32 | 1.08 | 5.65 | 12.99 | 9.14 | 0.81 | 6.98 | 11.29 |
| 0.35 | 8.93 | 1.08 | 5.27 | 12.58 | 9.23 | 0.82 | 7.06 | 11.41 |
| 0.36 | 9.09 | 1.07 | 5.44 | 12.73 | 9.34 | 0.85 | 7.09 | 11.60 |
| 0.37 | 9.42 | 1.06 | 5.81 | 13.03 | 9.46 | 0.88 | 7.12 | 11.79 |
| 0.38 | 9.57 | 1.05 | 6 | 13.15 | 9.56 | 0.90 | 7.15 | 11.97 |
| 0.39 | 9.62 | 1.08 | 5.97 | 13.28 | 9.68 | 0.95 | 7.15 | 12.20 |
| 0.4 | 9.94 | 1.11 | 6.16 | 13.73 | 9.80 | 1.01 | 7.11 | 12.48 |
| 0.41 | 10.28 | 1.14 | 6.4 | 14.16 | 9.94 | 1.09 | 7.05 | 12.84 |
| 0.42 | 10.1 | 1.14 | 6.22 | 13.97 | 10.07 | 1.14 | 7.03 | 13.10 |
| 0.43 | 10.01 | 1.11 | 6.24 | 13.78 | 10.18 | 1.17 | 7.07 | 13.29 |
| 0.44 | 10.21 | 1.06 | 6.59 | 13.82 | 10.18 | 1.19 | 7.00 | 13.35 |
| 0.45 | 10.23 | 1.02 | 6.78 | 13.68 | 10.22 | 1.20 | 7.02 | 13.42 |
| 0.46 | 10.29 | 0.98 | 6.97 | 13.61 | 10.26 | 1.23 | 7.00 | 13.53 |
| 0.47 | 10.4 | 0.98 | 7.08 | 13.71 | 10.36 | 1.27 | 6.99 | 13.73 |
| 0.48 | 10.42 | 1.01 | 7 | 13.83 | 10.46 | 1.29 | 7.02 | 13.90 |
| 0.49 | 10.32 | 1.03 | 6.83 | 13.81 | 10.56 | 1.31 | 7.07 | 14.04 |
| 0.5 | 10.38 | 1.07 | 6.75 | 14 | 10.65 | 1.34 | 7.07 | 14.23 |
| 0.51 | 10.55 | 1.14 | 6.68 | 14.42 | 10.71 | 1.34 | 7.13 | 14.29 |
| 0.52 | 10.81 | 1.22 | 6.66 | 14.96 | 10.77 | 1.34 | 7.19 | 14.35 |
| 0.53 | 10.72 | 1.27 | 6.42 | 15.02 | 10.72 | 1.33 | 7.19 | 14.25 |
| 0.54 | 10.3 | 1.25 | 6.04 | 14.56 | 10.63 | 1.31 | 7.14 | 14.12 |
| 0.55 | 10.06 | 1.17 | 6.08 | 14.05 | 10.56 | 1.29 | 7.12 | 13.99 |
| 0.56 | 10.34 | 1.14 | 6.47 | 14.22 | 10.56 | 1.28 | 7.15 | 13.98 |
| 0.57 | 10.34 | 1.14 | 6.47 | 14.22 | 10.67 | 1.27 | 7.30 | 14.04 |
| 0.58 | 10.68 | 1.15 | 6.78 | 14.59 | 10.87 | 1.27 | 7.49 | 14.24 |
| 0.59 | 11.34 | 1.17 | 7.35 | 15.33 | 11.09 | 1.26 | 7.75 | 14.44 |
| 0.6 | 11.61 | 1.17 | 7.63 | 15.59 | 11.34 | 1.30 | 7.88 | 14.80 |
| 0.61 | 11.9 | 1.13 | 8.07 | 15.73 | 11.49 | 1.33 | 7.95 | 15.04 |
| 0.62 | 12.38 | 1.1 | 8.65 | 16.11 | 11.63 | 1.37 | 7.98 | 15.28 |
| 0.63 | 12.59 | 1.09 | 8.87 | 16.3 | 11.73 | 1.45 | 7.86 | 15.59 |
| 0.64 | 12.33 | 1.1 | 8.6 | 16.06 | 11.78 | 1.48 | 7.85 | 15.71 |
| 0.65 | 11.87 | 1.08 | 8.22 | 15.53 | 11.80 | 1.51 | 7.78 | 15.83 |
| 0.66 | 11.9 | 1.02 | 8.43 | 15.38 | 11.79 | 1.52 | 7.74 | 15.83 |
| 0.67 | 12.06 | 1 | 8.68 | 15.44 | 11.77 | 1.49 | 7.79 | 15.75 |
| 0.68 | 11.88 | 1.02 | 8.43 | 15.33 | 11.73 | 1.49 | 7.78 | 15.69 |
| 0.69 | 11.96 | 1.06 | 8.36 | 15.57 | 11.73 | 1.48 | 7.80 | 15.66 |
| 0.7 | 12.13 | 1.1 | 8.41 | 15.85 | 11.72 | 1.46 | 7.82 | 15.62 |
| 0.71 | 11.92 | 1.12 | 8.11 | 15.72 | 11.72 | 1.46 | 7.84 | 15.60 |
| 0.72 | 11.62 | 1.17 | 7.63 | 15.6 | 11.73 | 1.44 | 7.89 | 15.57 |
| 0.73 | 11.82 | 1.23 | 7.65 | 16 | 11.80 | 1.42 | 8.01 | 15.58 |
| 0.74 | 12.3 | 1.24 | 8.09 | 16.5 | 11.80 | 1.37 | 8.14 | 15.46 |
| 0.75 | 12.42 | 1.21 | 8.32 | 16.51 | 11.79 | 1.35 | 8.20 | 15.39 |
| 0.76 | 12.39 | 1.16 | 8.45 | 16.32 | 11.80 | 1.32 | 8.28 | 15.32 |
| 0.77 | 12.1 | 1.14 | 8.22 | 15.98 | 11.75 | 1.23 | 8.47 | 15.03 |
| 0.78 | 12.02 | 1.19 | 7.99 | 16.06 | 11.67 | 1.17 | 8.56 | 14.78 |
| 0.79 | 11.94 | 1.25 | 7.69 | 16.19 | 11.56 | 1.10 | 8.63 | 14.49 |
| 0.8 | 11.53 | 1.31 | 7.08 | 15.98 | 11.43 | 1.03 | 8.70 | 14.17 |
| 0.81 | 11.48 | 1.33 | 6.96 | 16 | 11.29 | 0.98 | 8.68 | 13.90 |
| 0.82 | 11.38 | 1.33 | 6.87 | 15.89 | 11.12 | 0.89 | 8.74 | 13.49 |
| 0.83 | 10.81 | 1.36 | 6.19 | 15.43 | 10.97 | 0.77 | 8.93 | 13.02 |
| 0.84 | 10.26 | 1.4 | 5.52 | 15 | 11.02 | 0.67 | 9.23 | 12.81 |
| 0.85 | 10.16 | 1.37 | 5.5 | 14.83 | 10.98 | 0.66 | 9.23 | 12.73 |
| 0.86 | 10.55 | 1.28 | 6.21 | 14.9 | 10.84 | 0.66 | 9.07 | 12.61 |
| 0.87 | 10.71 | 1.15 | 6.81 | 14.61 | 10.77 | 0.68 | 8.95 | 12.60 |
| 0.88 | 10.48 | 1.02 | 7 | 13.95 | 10.74 | 0.65 | 9.01 | 12.47 |
| 0.89 | 10.51 | 0.95 | 7.29 | 13.73 | 10.83 | 0.63 | 9.14 | 12.52 |
| 0.9 | 11.09 | 0.91 | 8.01 | 14.17 | 10.96 | 0.61 | 9.33 | 12.59 |
| 0.91 | 11.31 | 0.87 | 8.35 | 14.27 | 11.19 | 0.64 | 9.49 | 12.88 |
| 0.92 | 11.32 | 0.85 | 8.42 | 14.22 | 11.25 | 0.60 | 9.64 | 12.85 |
| 0.93 | 11.46 | 0.87 | 8.52 | 14.41 | 11.25 | 0.67 | 9.47 | 13.03 |
| 0.94 | 11.68 | 0.91 | 8.6 | 14.76 | 11.23 | 0.78 | 9.16 | 13.29 |
| 0.95 | 12.04 | 0.98 | 8.71 | 15.36 | 11.23 | 0.73 | 9.28 | 13.19 |
| 0.96 | 11.89 | 1.09 | 8.19 | 15.58 | 11.15 | 0.81 | 8.99 | 13.31 |
| 0.97 | 11.49 | 1.13 | 7.66 | 15.31 | 10.76 | 0.91 | 8.33 | 13.20 |
| 0.98 | 10.71 | 1.19 | 6.67 | 14.76 | 10.34 | 1.04 | 7.56 | 13.12 |
| 0.99 | 10.68 | 1.66 | 5.06 | 16.3 | 10.24 | 1.64 | 5.88 | 14.60 |

**Appendix L**

**
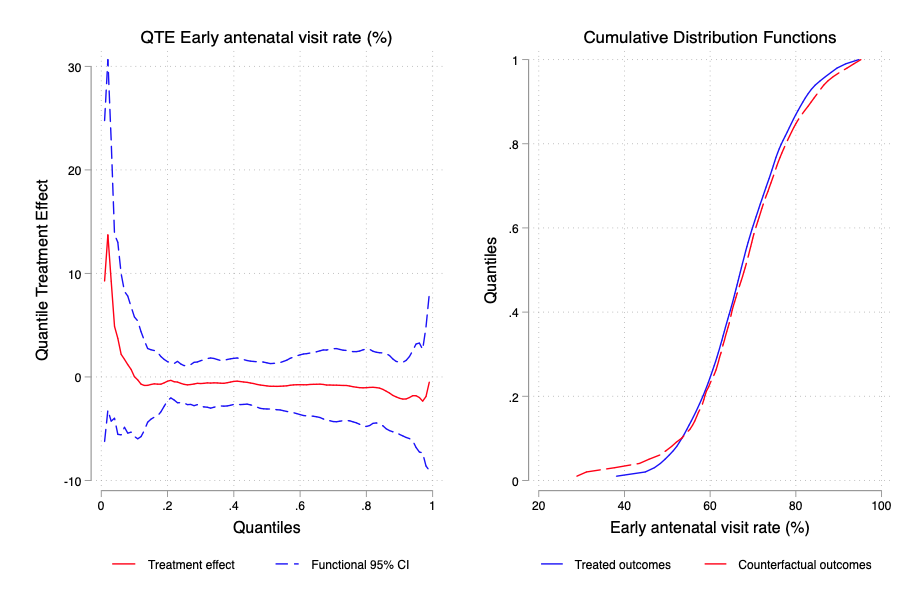
**

**
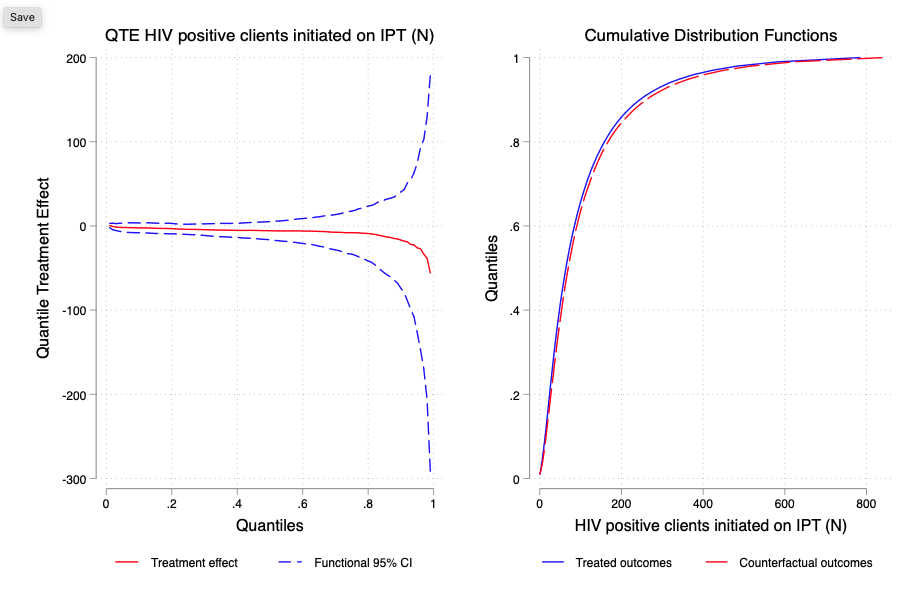
**

**
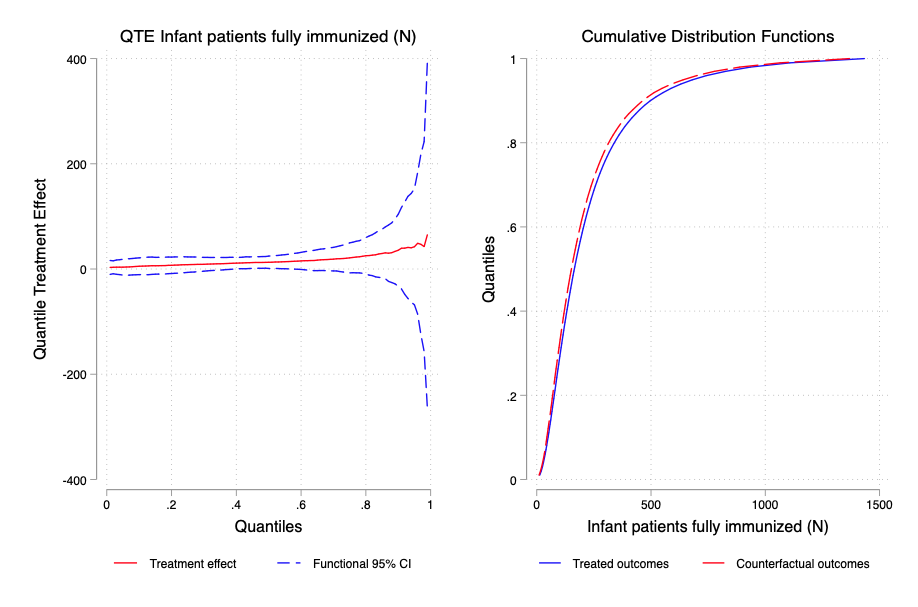
**

**
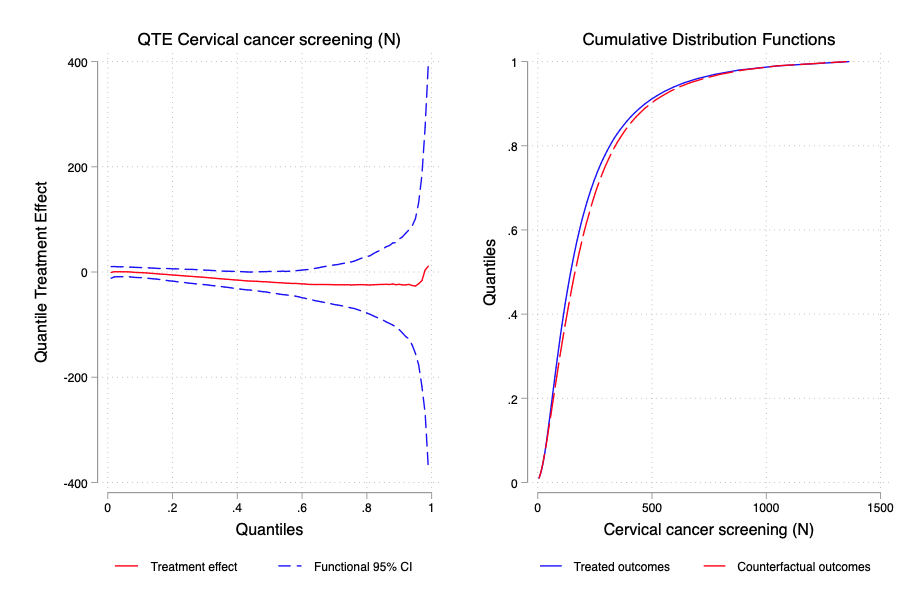
**

**Appendix M**

**Appendix N**

Rosenbaum and Rubin (1983) illustrated that if treatment assignment is unconfounded given covariates i.e. $Y_{i}\perp D|X$ then it is also unconfounded given the propensity score, $Y_{i}\perp D|p(X)$ where $p\left( X \right)=\Pr\left( D=1 | X \right)=E[D|X]$. We use probit regressions to estimate the propensity scores, which give the probability of enrolment in the ICRMP QI programme. First we estimate the propensity score using all the pre-treatment DHIS variable *levels* and covariates:

$$\Pr\left( D_{f}=1 | {DHIS}_{ft^{'},t^{'}<t^{*}},X_{ft^{*}} \right)=\theta\left( \alpha+\sum_{i=1}^{k} \sum_{j=1}^{t^{*}-1} \delta_{j}{DHIS}_{f,\left( t^{*}-j \right)}+\rho X_{ft^{*}} \right)$$

Where $\theta$ is the normal cumulative distribution function, $\alpha$ is the intercept, $\delta$ is the coefficients for the $j$ pre-treatment periods of the $k$ DHIS variables and $t^{*}$ is the enrolment period. We include four quarters of pre-treatment DHIS data capturing measures of facility activities from Q2 2014-Q2 2015 i.e. the year leading to QI programme enrolment^[[1]](#footnote-2)^. Probit regressions are run separately for the quartiles stratified by facilities baseline quality. We also run a specification using *trends* in facility activities instead of levels. In this case matching is based on the differences in DHIS metrics across quarters therefore matching ignores level differences and is based solely on changes in the performance of DHIS indicators.

$$\Pr\left( D_{f}=1 | \left( {DHIS}_{ft^{'},t^{'}<t^{*}}-{DHIS}_{f(t^{'}-1),t^{'}<t^{*}} \right),X_{ft^{*}} \right)=\theta\left( \alpha+\sum_{i=1}^{k} \sum_{j=1}^{t^{*}-2} \delta_{j}\left( {DHIS}_{f,\left( t^{*}-j \right)}-{DHIS}_{f,\left( t^{*}-j-1 \right)} \right)+\rho X_{ft^{*}} \right)$$

Estimated conditional probabilities of treatment i.e. propensity scores are generated from these models. **Appendix** **Table J1** presents the average marginal effects from the individual covariates for the model in *levels*. This allows exploration of the determinants of ICRMP enrolment for all facilities in addition to how determinants may vary when focusing on enrolment within strata defined by baseline quality.

In order for propensity scores – $p\left( X \right)=\Pr\left( D=1 | X \right)=E(D|X)$ – to be used to correctly estimate the ATT given as $E\left[ E\left( Y_{1f} | D_{f}=1, p\left( X_{f} \right) \right)-E\left( Y_{0f} | D_{f}=0, p\left( X_{f} \right) \right) | D_{f}=1 \right]$, we need the pre-treatment variables to be balanced given the propensity score such that: $D\perp X | p(X)$. Therefore, we check the balance of the propensity scores estimated from the probit models (outputs in table PS1). Following Becker & Ichino (2002) we check the balancing property after estimation of the propensity score. The balancing property is achieved in all but one covariate in 1 block for the 3^rd^ quartile and 5 covariates in 2 blocks in the 4^th^ quartile. Given the large number of covariates in the probit models this should still be sufficient to proceed with the analysis under the supposition that we have reduced (observable) selection bias such that the enrolled and non-enrolled facilities are sufficiently similar.

The above graphs show that overlap of the propensity scores between enrolled and non-enrolled facilities generally hold, although at the extremes of the distributions common support is not guaranteed.

We follow a procedure outlined by Heckman, Ichimura and Todd (1997) implementing a kernel propensity-score matching difference-in-difference estimator. Estimation augments the standard DD estimator, whereby instead of controlling for covariates, $X$, in a regression framework, each enrolled facility is matched to the whole sample of non-enrolled facilities based on weights defined by the propensity score. The weights are given by:

$$w_{f}=\frac{K\left( \frac{p\left( X \right)-p_{k}\left( X \right)}{h_{k}} \right)}{\sum K\left( \frac{p\left( X \right)-p_{k}\left( X \right)}{h_{k}} \right)}$$

Where $K(.)$ is the kernel function and $h_{n}$ is the bandwidth. We use a bandwidth of 0.08 as the smallest value for which all samples are able to provide an estimate. The re-weighted sample is then used to compute^[[2]](#footnote-3)^:

$${ATT}_{MAT-DiD}=\left[ E\left[ Y_{ft} | f=QI+,t=2016 \right]-w_{f}*E\left[ Y_{ft} | f=QI+,t=2015 \right] \right]-\left[ E\left[ Y_{ft} | f=QI-,t=2016 \right]-w_{f}*E\left[ Y_{ft} | f=QI-,t=2015 \right] \right]$$

| **Table J1: Probit estimation of ICRMP enrolment** | | | | | |
| --- | --- | --- | --- | --- | --- |
|  | Full sample | Lowest baseline quality | Low baseline quality | High baseline quality | Highest baseline quality |
| District | -0.0126*** | -0.0160*** | -0.0258*** | -0.0153*** | -0.00272 |
|  | (0.00238) | (0.00588) | (0.00529) | (0.00521) | (0.00515) |
| Municipality proportion with no schooling | -2.768* | -2.824 | 1.370 | -9.116*** | -0.556 |
|  | (1.495) | (3.667) | (3.257) | (3.426) | (2.891) |
| Municipality proportion population urban dwelling | 0.953** | 1.091 | 1.694* | 1.319 | 1.482* |
|  | (0.405) | (1.039) | (0.884) | (0.861) | (0.864) |
| Municipality average household size | -0.0108 | -0.952*** | -0.471 | 0.383 | 0.389* |
|  | (0.124) | (0.355) | (0.292) | (0.264) | (0.232) |
| Municipality proportion households with flush toilet | -0.407 | -1.055 | -1.902* | -0.517 | -0.494 |
|  | (0.527) | (1.298) | (1.155) | (1.104) | (1.219) |
| Municipality proportion households with piped water | -0.915*** | -1.497** | -0.487 | -1.786*** | -0.226 |
|  | (0.280) | (0.636) | (0.614) | (0.625) | (0.610) |
| South African Index of Multiple Deprivation (quintiles) | -0.120*** | -0.218** | -0.0230 | -0.201** | -0.123* |
|  | (0.0381) | (0.111) | (0.0880) | (0.0786) | (0.0736) |
| Distance to closest facility | 0.0340*** | 0.0368* | 0.0531*** | 0.0291** | 0.0174 |
|  | (0.00785) | (0.0210) | (0.0199) | (0.0140) | (0.0152) |
| Number of facilities within 10km | 0.0536*** | 0.129*** | 0.0581** | 0.0662** | 0.0247 |
|  | (0.0133) | (0.0352) | (0.0291) | (0.0293) | (0.0275) |
| Population within 10km | -0.00000139*** | -0.00000297*** | -0.00000104 | -0.00000192** | -0.000000907 |
|  | (0.000000315) | (0.000000799) | (0.000000651) | (0.000000755) | (0.000000695) |
| Q3 2014: Average monthly children <1 years fully immunised for quarter | 0.00262 | 0.00525 | 0.00997 | 0.0000898 | 0.000382 |
|  | (0.00356) | (0.0112) | (0.00937) | (0.00963) | (0.00537) |
| Q3 2014: Average monthly patient head count | 0.000108 | -0.000196 | 0.000325 | 0.000236 | 0.000279 |
|  | (0.0000951) | (0.000265) | (0.000277) | (0.000264) | (0.000225) |
| Q3 2014: Average monthly patients seen by professional nurse | -0.0000265 | 0.0000287 | -0.000326 | -0.000133 | 0.0000228 |
|  | (0.000104) | (0.000257) | (0.000293) | (0.000272) | (0.000242) |
| Q3 2014: Average monthly professional nurse days at facility | 0.000338 | 0.00168 | 0.00327 | 0.00138 | -0.00435 |
|  | (0.000449) | (0.00197) | (0.00242) | (0.00109) | (0.00372) |
| Q3 2014: Average monthly rate of ANC 1st visit before 20 weeks | -0.00259 | 0.00450 | -0.00554 | -0.00664 | -0.00848 |
|  | (0.00296) | (0.00698) | (0.00621) | (0.00604) | (0.00650) |
| Q3 2014: Average monthly number of cervical cancer screenings >30 years | 0.000918 | 0.00533 | -0.00155 | 0.00621 | -0.000413 |
|  | (0.00288) | (0.00799) | (0.00690) | (0.00654) | (0.00648) |
| Q3 2014: Average monthly number of RV 2nd doses for <1 years | -0.00493 | 0.00201 | 0.00393 | 0.000913 | -0.0290** |
|  | (0.00507) | (0.0116) | (0.0128) | (0.0113) | (0.0114) |
| Q3 2014: Average monthly number of HIV+ new client initiated on IPT | -0.00413 | 0.0115 | 0.00588 | -0.0133* | -0.00379 |
|  | (0.00353) | (0.0122) | (0.00968) | (0.00744) | (0.00728) |
| Q3 2014: Average monthly tracer item stockout rate | 0.00296** | -0.00168 | 0.00466 | 0.00337 | 0.00711** |
|  | (0.00130) | (0.00309) | (0.00293) | (0.00250) | (0.00297) |
| Q4 2014: Average monthly children <1 years fully immunised for quarter | 0.000586 | 0.0128 | -0.00379 | 0.0219* | -0.0191* |
|  | (0.00543) | (0.0157) | (0.0139) | (0.0127) | (0.0101) |
| Q4 2014: Average monthly patient head count | 0.0000268 | 0.000434 | -0.000627* | -0.000216 | 0.0000338 |
|  | (0.000109) | (0.000327) | (0.000358) | (0.000306) | (0.000241) |
| Q4 2014: Average monthly patients seen by professional nurse | -0.0000623 | -0.000260 | 0.000735* | 0.000191 | -0.000431* |
|  | (0.000112) | (0.000317) | (0.000377) | (0.000307) | (0.000242) |
| Q4 2014: Average monthly professional nurse days at facility | -0.0000431 | -0.000725 | -0.0000304 | -0.00356 | 0.00768 |
|  | (0.000393) | (0.00223) | (0.000453) | (0.00315) | (0.00516) |
| Q4 2014: Average monthly rate of ANC 1st visit before 20 weeks | -0.00236 | 0.00435 | -0.00555 | 0.00104 | -0.0125* |
|  | (0.00315) | (0.00748) | (0.00692) | (0.00667) | (0.00638) |
| Q4 2014: Average monthly number of cervical cancer screenings >30 years | -0.00158 | -0.00718 | -0.00241 | -0.00371 | 0.00359 |
|  | (0.00319) | (0.00954) | (0.00623) | (0.00763) | (0.00780) |
| Q4 2014: Average monthly number of RV 2nd doses for <1 years | 0.00000472 | -0.00779 | -0.0104 | -0.00737 | 0.0278** |
|  | (0.00610) | (0.0156) | (0.0145) | (0.0141) | (0.0133) |
| Q4 2014: Average monthly number of HIV+ new client initiated on IPT | 0.00881** | 0.0187 | 0.0149 | 0.0132 | 0.00130 |
|  | (0.00420) | (0.0126) | (0.0134) | (0.00883) | (0.00823) |
| Q4 2014: Average monthly tracer item stockout rate | -0.000502 | 0.000409 | 0.00173 | -0.00334 | -0.000738 |
|  | (0.00136) | (0.00338) | (0.00310) | (0.00261) | (0.00301) |
| Q1 2015: Average monthly children <1 years fully immunised for quarter | 0.00442 | -0.00115 | -0.0117 | 0.00466 | 0.0180 |
|  | (0.00516) | (0.0145) | (0.0154) | (0.00981) | (0.0122) |
| Q1 2015: Average monthly patient head count | 0.00000537 | -0.000101 | 0.000353 | 0.0000324 | 0.0000872 |
|  | (0.000101) | (0.000239) | (0.000368) | (0.000226) | (0.000232) |
| Q1 2015: Average monthly patients seen by professional nurse | -0.0000666 | -0.0000868 | -0.000554* | 0.000105 | 0.000158 |
|  | (0.000103) | (0.000271) | (0.000318) | (0.000233) | (0.000235) |
| Q1 2015: Average monthly professional nurse days at facility | -0.000117 | 0.00261 | -0.000893 | 0.000204 | -0.00606* |
|  | (0.000855) | (0.00302) | (0.00137) | (0.00297) | (0.00360) |
| Q1 2015: Average monthly rate of ANC 1st visit before 20 weeks | -0.00213 | -0.00773 | -0.00859 | -0.00193 | 0.00387 |
|  | (0.00314) | (0.00797) | (0.00694) | (0.00640) | (0.00636) |
| Q1 2015: Average monthly number of cervical cancer screenings >30 years | 0.00287 | -0.00503 | 0.00869 | 0.00725 | 0.00409 |
|  | (0.00325) | (0.00932) | (0.00880) | (0.00660) | (0.00723) |
| Q1 2015: Average monthly number of RV 2nd doses for <1 years | -0.00721 | -0.0259* | 0.00629 | -0.0209 | -0.00153 |
|  | (0.00562) | (0.0155) | (0.0144) | (0.0143) | (0.0107) |
| Q1 2015: Average monthly number of HIV+ new client initiated on IPT | -0.00642* | -0.0330*** | -0.0235** | 0.0119 | -0.00406 |
|  | (0.00359) | (0.0116) | (0.0118) | (0.00780) | (0.00610) |
| Q1 2015: Average monthly tracer item stockout rate | -0.00122 | 0.000333 | -0.00134 | -0.000135 | -0.00262 |
|  | (0.00133) | (0.00348) | (0.00297) | (0.00268) | (0.00286) |
| Q2 2015: Average monthly children <1 years fully immunised for quarter | 0.00995** | 0.0119 | 0.0183 | -0.00442 | 0.0156 |
|  | (0.00500) | (0.0138) | (0.0162) | (0.00864) | (0.0108) |
| Q2 2015: Average monthly patient head count | -0.000103 | -0.0000883 | -0.000122 | 0.0000536 | -0.000208 |
|  | (0.0000790) | (0.000246) | (0.000207) | (0.000186) | (0.000186) |
| Q2 2015: Average monthly patients seen by professional nurse | 0.000186** | 0.000364 | 0.000277 | -0.000187 | 0.000141 |
|  | (0.0000792) | (0.000231) | (0.000180) | (0.000196) | (0.000193) |
| Q2 2015: Average monthly professional nurse days at facility | 0.000653 | -0.000686 | -0.000438 | 0.00255 | 0.000477 |
|  | (0.000696) | (0.00175) | (0.00175) | (0.00198) | (0.00151) |
| Q2 2015: Average monthly rate of ANC 1st visit before 20 weeks | 0.00708** | 0.0105 | 0.0131** | 0.00588 | 0.00181 |
|  | (0.00277) | (0.00684) | (0.00623) | (0.00540) | (0.00584) |
| Q2 2015: Average monthly number of cervical cancer screenings >30 years | -0.000390 | -0.000467 | -0.00465 | -0.000674 | 0.000658 |
|  | (0.00250) | (0.00857) | (0.00858) | (0.00362) | (0.00660) |
| Q2 2015: Average monthly number of RV 2nd doses for <1 years | -0.00320 | 0.00516 | -0.00877 | -0.0000867 | -0.0154 |
|  | (0.00542) | (0.0146) | (0.0145) | (0.0113) | (0.0115) |
| Q2 2015: Average monthly number of HIV+ new client initiated on IPT | -0.00235 | 0.0147 | -0.000153 | -0.0115 | -0.00133 |
|  | (0.00346) | (0.0118) | (0.0125) | (0.00764) | (0.00539) |
| Q2 2015: Average monthly tracer item stockout rate | 0.00169 | 0.00236 | 0.00165 | 0.00199 | 0.00410* |
|  | (0.00111) | (0.00277) | (0.00245) | (0.00228) | (0.00242) |
| Constant | 0.741 | 3.900** | 1.882 | 0.877 | -0.400 |
|  | (0.579) | (1.650) | (1.369) | (1.241) | (1.072) |
| N | 1,633 | 361 | 392 | 423 | 457 |

**Appendix A Cont.**

| **Components** | **Subcomponents** | **Elements** |
| --- | --- | --- |
| **Component 1: Administration** | Subcomponent 1: Signage and Notices | All way-finding signage in place |
|  |  | Facility information board reflects the facility name, service hours, physical address, contact details for facility and emergency service and service package details is visibly displayed at the entrance of the premises |
|  |  | Sign indicating NO WEAPONS, NO SMOKING, NO ANIMALS (except for service animals), NO LITTERING and NO HAWKERS is clearly sign posted at the entrance of the facility |
|  |  | The Vision, Mission and Values of the district must be visibly displayed |
|  |  | Facility organogram with contact details of the facility manager is displayed on a central notice board |
|  |  | All service areas within the facility are clearly signposted |
|  | Subcomponent 2: Staff Identity and Dress Code | There is a prescribed dress code for all service providers |
|  |  | All staff members comply with prescribed dress code |
|  | Subcomponent 3: Client Services Organisation | A functional wheelchair is available |
|  |  | Staff are scheduled such that helpdesk/reception services are available |
|  |  | There is a process that prioritizes the very sick, frail and elderly patients |
|  |  | There is access for people in wheelchairs |
|  | Subcomponent 4: Management of Client Records | Patient record content adheres to ICSM prescripts |
|  |  | Patient records are filed in close proximity to patient registration desk |
|  |  | Priority stationery (clinical and administrative) is available at the facility in sufficient quantities |
|  |  | The guideline for filing, archiving and disposal of patient records is adhered to |
|  |  | The provincial/district Standard Operating Procedure/guideline for filing, archiving and disposal of patient records is available |
|  |  | The retrieval of a patient ’s file takes less than ten minutes |
|  |  | There is a single location for storage of all active patient records |
|  |  | There is a single patient record (except for active TB patients) irrespective of health conditions |
| **Component 2: Integrated Clinical Services Management** | Subcomponent 5: Clinical Services Provision | Ante-natal patient initiated on ART rate is at least 95% or has increased by at least 5% from the previous year |
|  |  | Ante-natal visit rate before 20 weeks gestation is at least 62% or has increased by at least 5% from the previous year |
|  |  | Immunisation coverage under one year (annualised) is at least 92 % or has increased by at least 5% from the previous year |
|  |  | TB (new pulmonary) defaulter rate < 5% |
|  |  | TB treatment success rate is at least 85% or has increased by at least 5% from the previous year |
|  |  | The facility has been reorganised with designated consulting areas and staffing for acute, chronic health conditions and preventative health services. |
|  | Subcomponent 7: Management of Client Appointments | An ICSM compliant patient appointment system for patients with stabilised chronic health conditions and MCWH patient is in use |
|  |  | The records of booked patients are pre retrieved at least 24 hours before the appointment |
|  |  | Pre-dispensed medication for clinically stable chronic patients is prepared for collection 24 hours prior to collection date/ or patients are enrolled on the CCMDD programme |
|  | Subcomponent 8: Coordination of PHC Services | Facility renders school health services to schools in its catchment areas |
|  |  | The facility refers patients with chronic but stable health conditions to home- and community-care services (WBPHCOT) for support |
|  | Subcomponent 9: Clinical Guidelines and Protocols | The ICSM compliant package of clinical guidelines is available in all consulting rooms |
|  |  | 80% of professional nurses have been fully trained on ICSM compliant package of clinical guidelines |
|  |  | 80% of Professional Nurses have been trained on Basic Life Support |
|  |  | The National Clinical Audit guideline is available |
|  | Subcomponent 10: Infection Prevention and Control | Sharps are disposed of in impenetrable, tamperproof containers |
|  |  | Sharps containers are disposed of when they reach the limit mark |
|  |  | Sharps containers are placed on work surface or in wall mounted brackets |
|  |  | Staff wear appropriate protective clothing |
|  |  | The National Policy on Infection Prevention and Control is available |
|  |  | The linen in use is clean |
|  |  | The linen is appropriately used for its intended purpose |
|  |  | Waste is properly segregated |
|  |  | There is a staff member who is assigned infection prevention and control role in a facility |
|  | Subcomponent 11: Client Waiting Time | The average time that a patient spend in the facility is not longer than 3 hours |
|  |  | The standard waiting time for every service area is visibly posted |
|  |  | Patients are intermittently informed of delays and reasons for delays in service provision |
|  |  | The National policy for the management of waiting times is available |
|  |  | Waiting time is monitored using the prescribed tool |
|  | Subcomponent 12: Patient Experience of Care | Official complaint/compliment/suggestion forms and pen are available |
|  |  | Complaints/compliments/suggestions boxes are visibly placed at main entrance/exit |
|  |  | The National Patient Experience of Care guideline is available |
|  |  | The National Policy to manage Complaints/Compliments/Suggestions is available |
|  |  | A standardised poster describing the process to follow to lodge a complaint, give a compliment or make a suggestion is clearly sign posted next to the complaints/compliments/suggestions box |
|  |  | The complaint/compliments/suggestions records show compliance to the National Policy to manage Complaints/Compliments/Suggestions |
|  |  | An average overall score of 60% is obtained in the patient experience of care survey. |
|  |  | The facility's/district's Standard Operating Procedure to manage Complaints/Compliments/Suggestions is available |
|  |  | The results of the yearly Patient Experience of Care survey are visibly displayed at reception |
|  |  | The results obtained from the Patient experience of Care survey are used to improve the quality of service provision |
| **Component 3: Medicines, Supplies and Laboratory Services** | Subcomponent 13: Medicines and Supplies | The temperature of the medicine refrigerator is maintained within the safety range |
|  |  | The temperature of the medicine refrigerator is recorded twice daily |
|  |  | There is at least one functional wall mounted room thermometer in the medicine room/dispensary |
|  |  | The temperature of the medicine room/dispensary is maintained within the safety range |
|  |  | The temperature of the medicine room/dispensary is recorded daily |
|  | Subcomponent 14: Management of Laboratory | Required functional diagnostic equipment and concurrent consumables for point of care testing are available |
|  |  | The Primary Health Care Laboratory Handbook is available |
|  |  | Specimens are collected, packaged, stored and prepared for transportation according to the Primary Health Care Laboratory Handbook |
|  |  | The laboratory results are received from the laboratory within the specified turn around times |
| **Component 4: Human Resources for Health** | Subcomponent 15: Staff Allocation and Use | An annual leave schedule is available |
|  |  | A facility with a work-load of more than 150 patients per day has a dedicated facility manager whose work content consists of approximately 80% management and 20% clinical work |
|  |  | Leave policy is available |
|  |  | Staffing is in line with WISN |
|  |  | Staffing needs have been determined in line with WISN |
|  |  | Work allocation schedule is signed by all staff members |
|  | Subcomponent 16: Professional Standards and Performance Management Development System (PMDS) | Staff satisfaction survey is conducted annually |
|  |  | The grievance procedure is available |
|  |  | The disciplinary procedure is available |
|  |  | The results of the staff satisfaction survey is used to improve the work environment |
|  |  | Continued staff development needs are determined for the current financial year and submitted to the district manager |
|  |  | There is an individual Performance Management Agreement for each staff member |
|  |  | Training records reflect planned training is conducted as per the district training programme |
|  | Subcomponent 6: Availability of Medical, Mental health and Allied health practitioners | Patients have access to a medical practitioner |
|  |  | Patients have access to dietetic services |
|  |  | Patients have access to mental health services |
|  |  | Patients have access to oral health services |
|  |  | Patients have access to occupational therapy services |
|  |  | Patients have access to physiotherapy services |
|  |  | Patients have access to ophthalmic service |
|  |  | Patients have access to radiography services |
|  |  | Patients have access to speech and hearing services |
|  |  | Patients have access to social work services |
| **Component 5: Support Services** | Subcomponent 18: Hygiene and Cleanliness | All service areas are clean |
|  |  | All cleaners have been trained on cleaning |
|  |  | A signed waste removal service level agreement between the health department and the service provider is available |
|  |  | Clean running water, toilet paper, liquid hand wash soap and disposable hand paper towels are available |
|  |  | All toilets are clean, intact and functional |
|  |  | All work completed is signed off by cleaners |
|  |  | General disposal bins are lined with transparent or black plastic bin liners and have functional lids |
|  |  | Cleaning materials are available |
|  |  | Sanitary and health care waste disposal bins are lined with red plastic bin liners and have functional lids |
|  |  | The exterior of the facility is aesthetically pleasing and clean |
|  |  | Waste is stored in access-controlled rooms |
|  |  | Waste is removed in line with the contract |
|  | Subcomponent 19: Security | There is a standard security guard room |
|  |  | Perimeter fencing is intact |
|  |  | A signed copy of the service level agreement between the security company and the provincial department of health is available |
|  | Subcomponent 20: Outbreak and Disaster Preparedness | Functional firefighting equipment is available |
|  |  | Deficiencies identified during the practice of the emergency evacuation drill are addressed |
|  |  | The emergency evacuation procedure is practiced annually |
| **Component 6: Infrastructure** | Subcomponent 21: Physical Space and Routine | Clinic space accommodates all services and staff |
|  | Subcomponent 22: Essential Equipment and Furniture | There is a sterile emergency delivery pack |
|  |  | Furniture is available and intact in service areas |
|  |  | Restore the emergency trolley daily or after every time it was used |
|  |  | Oxygen cylinder with pressure gauges available in resuscitation/emergency room |
|  |  | Redundant and non-functional equipment is removed from the facility |
|  |  | Resuscitation room is equipped with functional basic equipment for resuscitation |
|  |  | Essential equipment is available and functional in every consulting room |
|  | Subcomponent 23: Bulk Supplies | The sewerage system is functional |
|  |  | The back-up electrical power supply is checked weekly to determine its functionality |
|  |  | There is constant supply of clean, running water to the facility |
|  |  | There is emergency water supply in the facility |
|  |  | There is functional back-up electrical supply |
|  | Subcomponent 24: ICT Infrastructure and Services | There is functional computer |
|  |  | There is a functional telephone in the facility |
|  |  | There is functional printer connected to the computer |
|  |  | There is web access |
| **Component 7: Health Information Management** | Subcomponent 25: District Health Information | District Health Information Management System policy available |
|  |  | There is a functional computerized patient information system |
|  |  | Relevant DHIS registers are available and are kept up to date |
| **Component 8: Communication** | Subcomponent 26: Internal Communication | There is at least a quarterly staff meeting held within the facility |
|  |  | There are sub district/district quarterly facility performance review meetings |
|  |  | Staff members demonstrate that incoming policies and notices have been read and are understood by appending their signatures on such policies and notifications |
|  | Subcomponent 27: Community Engagement | The facility has an annual open day |
|  |  | Contact details of clinic committee members are visibly displayed |
|  |  | There is a functional clinic committee |
| **Component 9: District Health System Support** | Subcomponent 28: District Health Support | The district Permanent Perfect Team for Ideal Clinic Realisation and Maintenance visits the clinic at least twice a year to record the Ideal Clinic Realization status and to correct weaknesses |
|  |  | There is a health facility operational plan in line with district health plan |
|  | Subcomponent 29: Planned and Emergency Patient Transport | EMS respond according to the pre-determined response time |
|  |  | There is a pre-determined EMS response time to the facility |
|  | Subcomponent 30: Referral System | The National Referral Policy is available |
|  |  | The facility's Standard Operating Procedure for referrals is available and sets out clear referral pathways |
|  |  | There is a referral register that records referred patients |
| **Component 10: Implementing Partners and Stakeholders** | Subcomponent 31: Implementing Partners Support | There is an up to date list (with contact details) of all implementing health partners that support the facility is available |
|  |  | The list of implementing health partners shows their areas of focus and business activities |
|  | Subcomponent 32: Multi-sectoral Collaboration | There is an official Memorandum of Understanding between the PDOH and Department of Public Works |
|  |  | There is an official Memorandum of Understanding between the PDOH and the Department of Social Development |
|  |  | There is an official Memorandum of Understanding between the NDOH and Department of Home Affairs |
|  |  | There is an official Memorandum of Understanding between the PDOH Department of Education |
|  |  | There is an official Memorandum of Understanding between the PDOH and SAPS |
|  |  | There is an official Memorandum of Understanding between the PDOH and department of transport |
|  |  | There is an official Memorandum of Understanding between the district management and Cooperative Governance and Traditional Affairs (CoGTA) |

**Supplementary Online Material**

**Supplementary Material A: Heterogeneous treatment effects**

There is not a single literature examining heterogeneous treatment effects, and there are many perspectives from which to examine the concept which spiral out to further topics with significant methodological implications^[[3]](#footnote-4)^. For ease of exposition, we specify two different but related concepts of treatment effect heterogeneity: (i) impact heterogeneity and (ii) essential heterogeneity. Methodologically, the former may be thought of primarily as an issue of functional form specification, while the latter can be more involved to address.

In order to dissect heterogeneous treatment effects we first lay out the standard model of homogenous effects assumed in policy evaluation with a binary treatment variable:

$$Y_{i}=\beta_{1}D_{i}+\beta_{2}X_{i} (1)$$

The describes the underlying data generating process (DGP) of the outcome variable. $Y_{i}$ is the observed outcome with $D_{i}=1$ if the observation is treated and $D_{i}=0$ if untreated and $X_{i}$ are observed confounders. This is the case of homogenous treatment effects as coefficient $\beta_{1}$ is assumed constant and fixed across all individuals. Equation $(1)$ may be estimated by OLS: $Y_{i}=\beta_{0}+\beta_{1}D_{i}+\beta_{2}X_{i}+\varepsilon_{i}$. It has been shown that, should the homogeneous treatment effect assumption, $\beta_{1}=ATE$. For this reason, $(1)$ has been referred to as the common-impact model as the $ATE$ is a scalar constant (Ravallion, 2008).

As mentioned, it is very common that the effect of a treatment on an outcome, may depend on the background characteristics of the treated unit. Impact heterogeneity occurs when treatment effects vary on the basis of observed pre-treatment characteristics. These treatment effect differences may be uncorrelated with treatment adoption. A reason, commonly encountered in epidemiology, is the occurrence of a ‘natural’ interaction which may be unknown to the individuals/units or agents allocating treatment. Alternatively, when treatment assignment is non-random or in RCTs with selective take-up, treatment adoption may be based by rational decisions of individuals or agents who allocate treatment based on perceived unit level treatment effects. In the former case, $\beta_{1}$ in equation $(1)$ would still represent the $ATE$. However, this would not hold if treatment allocation was correlated with treatment effect.

In both these circumstance, equation $(1)$ no longer accurately reflects the true DGP, as the relationship between the outcome and treatment depends on a moderating variable i.e. the treatment effect varies with the level of a pre-treatment covariate:

$$Y_{i}=\beta_{1}D_{i}(Z_{i})+\beta_{2}X_{i} (2)$$

The effect of the treatment on the outcome depends on $Z$, an observed sub-set of $X$. Now, $\beta_{1}$, the treatment effect depends on $Z_{i}$. This is commonly explored in the form of interaction or sub-group analysis, through the inclusion of treatment-covariate interactions in estimations. $Y_{i}=\beta_{0}+\beta_{1}D_{i}+\beta_{2}Z_{i}+\beta_{3}\left( D_{i}Z_{i} \right)+\beta_{4}X_{i}+\varepsilon_{i}$. Such multiplicative interaction models capture the variation in treatment effects allowing the $ATE$ to vary by sub-populations. In this case $ATE\neq\mathrm{ATE}(Z)$. $\beta_{1}$ and $\beta_{3}$ are estimates of the conditional average treatment effects ($CATE$), the (homogeneous) treatment effects given a subset of observables. Hypothesis testing of the $CATE$ allows for assessment of whether the average effect of treatment differs across sub-groups. However, even in cases where the $CATE$ is found to be equal, this does not necessarily suggest no treatment effect heterogeneity as there may still be distributions of treatment effects within each sub-group which averages to similar values.

Despite the increased flexibility of allowing the treatment effect to vary across sub-groups this can still be thought of as an augmented common-impact model. This is because although the specification allows the treatment effect to vary across the sub-groups specified by $Z_{i}$, it still assumes a constant treatment effect within those sub-groups.

It has been noted that there may be circumstances where the treatment effect varies even after accounting for $Z_{i}$, what has been referred to as ‘essential heterogeneity’ (Heckman et al. 2006)^[[4]](#footnote-6)^. In this circumstance the DGP may be represented as:

$$Y_{i}=\beta_{0}+\beta_{1i}D_{i}+\beta_{2}X_{i} (3)$$

The subscript $i$ on $\beta_{1}$ signifies the presence of treatment effect heterogeneity with $\beta_{1}$ no longer a fixed parameter. In this circumstance, $\beta_{1}$ varies even after controlling for $X_{i}$. Now, if we estimate this with a model assuming homogenous treatment effects i.e. $Y_{i}=\beta_{0}+\beta_{1}D_{i}+\beta_{2}X_{i}+\varepsilon_{i}$ (so we assume $\beta_{1i}=\beta_{1}$) our model is misspecified. Heckman et al. (2006) notes that when there are heterogeneous treatment effects and individuals or treatment allocating agents have some knowledge of the idiosyncratic benefits, this opens the possibility of sorting on the gain or selection on idiosyncratic unobservables. Such a circumstance would result in $Cov(\beta_{1}, D)\neq0$. It is clear to see that most often $Cov\left( \beta_{1}, D \right)>0$, with those with higher expected gains more likely to select or be selected into treatment. It can be seen that this issue is therefore related to but distinct from selection bias, $Cov(X_{i}, D_{i})\neq0$, which is sorting on the levels^[[5]](#footnote-7)^. Unlike impact heterogeneity, occurrence of essential heterogeneity implies that $ATE\neq ATT\neq ATU$.

It has been shown that in the presence of essential heterogeneity, linear regression models specified under the assumption of homogeneous treatment effects do not return estimates of the ATE, even if the selection on observables / ignorability assumption holds (Rhodes, 2010; Słoczyński, 2015). It is straight-forward to see how this issue carries through and can impact circumstances where the DGP is a combination of equations $(2)$ and $(3)$: $Y_{i}=\beta_{1i}D_{i}+\beta_{2}Z_{i}+\beta_{3i}\left( D_{i}Z_{i} \right)+\beta_{4}X_{i}$.

Exploration of impact heterogeneity is increasingly recognised as an important part of randomised controlled trials (Imai & Ratkovic, 2013). This is usually undertaken by exploring treatment-covariate interactions although more sophisticated methods are being developed (REFS). Analysis of impact heterogeneity is arguably still not undertaken as frequently as it should be in observational data, given the potential to examine policy-relevant issues. One reason for this may be the inability to pre-register analysis with observational data and the non-trivial risk of type I error when sub-groups are chosen post-hoc (Imai & Strauss, 2011).

Crump et al. (2008) developed two non-parametric tests of treatment effect heterogeneity. The first assesses whether a treatment has zero average effect for all sub-groups defined by covariates i.e. all conditional average treatment effects (CATEs) are zero. If $\gamma$ is a treatments effect and $X$ observed covariates, the test examines $\gamma\left( x \right)=0$ versus $\gamma\left( x \right)\neq0$. The second assesses whether the CATE is constant for all sub-groups i.e. there is no impact heterogeneity. Specifically assessing if $\gamma\left( x \right)=\gamma$ versus $\gamma\left( x \right)\neq\gamma$.

**References**

O’Neil, S. Kreif, N. Grieve, R. Sutton, M. Sekhon, J. (2016). Estimating causal effects: considering three alternatives to difference-in-differences estimation. *Health Services & Outcomes Research Methods.* 16. 1-21.

Giesselmann, M. Schmidt-Catran, A. (2018). Interactions in Fixed Effects Regression Models. *DIW Berlin Discussion Papers.*

Melly, B. Santangelo, G. (2015). The changes-in-changes model with covariates. Working Paper.

Rosenbaum, P. Rubin, D. (1983). The central role of the propensity score in observational studies for causal effects. *Biometrika.* Vol. 70. No. 1. 41-55.

Crump, R. Hotz, V. Imbens, G. Mitnik, O. (2008). Nonparametric tests for treatment effect heterogeneity. *Review of Economics and Statistics.* 90. 389-405.

Rhodes, W. (2010). Heterogeneous Treatment Effects: What Does a Regression Estimate? *Evaluation Review.* 34(4). 334-361.

Leuven, E. Sianesi, B. (2003). PSMATCH2: Stata module to perform full Mahalanobis and propensity score matching. Common support graphing and covariate imbalance testing. Statistical Software Components S432001, Boston College Department of Economics, revised Feb 2018.

Villa, J. (2009). DIFF: Stata module to perform Difference in Difference estimation. Statistical Software Components. Boston College Department of Economics. Revised 31 Dec 2019.

Becker, S. Ichino, A. (2002). Estimation of average treatment effects based on propensity scores. *Stata Journal.* Vol. 2. No. 4. 358-377.

Heckman, J. Ichimura, H. Todd, P. (1997). Matching as an Econometric Evaluation Estimator: Evidence from Evaluating a Job Training Programme. *The Review of Economic Studies.* Vol. 64. No. 4. 605-654.

Heckman, J. Urzua, S. Vytlacil, E. (2006). Understanding Instrumental Variables in Models with Essential Heterogeneity. *Review of Economics and Statistics.* Vol. LXXXVIII. No. 3.

Imai, K. Ratkovic, M. (2013). Estimating Treatment Effect Heterogeneity in Randomised Programme Evaluation. *The Annals of Applied Statistics*. Vol. 7. No. 1. 443-470.

Słoczyński, T. (2015). New Evidence on Linear Regression and Treatment Effect Heterogeneity, IZA Discussion Papers, No. 9491, Institute for the Study of Labor (IZA), Bonn.

Ravallion, M. (2008). Evaluation in the Practise of Development. *World Bank Policy Paper.*

Imai, K. Strauss, A. (2011). Estimation of Heterogeneous Treatment Effects from Randomized Experiments, with Application to the Optimal Planning of the Get-Out-the-Vote Campaign. *Political Analysis.* 19:1. 1-19.

1. We don’t include all quarterly data from January 2013 due to estimation issues with including such a large amount of variables in propensity score calculation in the semi-parametric approach used. [↑](#footnote-ref-2)
2. PSCORE by Becker & Ichino (2002) used to calculate propensity scores and undertake initial balance assessments. The matching algorithm is implemented using the PSMATCH2 Stata package by Leuven and Sianesi (2003). Combined Kernel propensity score matching with DiD is implemented using DIFF Stata package by Villa (2009). [↑](#footnote-ref-3)
3. Many different terminologies have been employed in this area including but not limited to sub-group analysis, heterogeneous treatment effects, interaction effects, distributional impact analysis, moderation analysis, effect modification, conditional average treatment effect estimation. [↑](#footnote-ref-4)
4. This has also been called the random-coefficient case, correlated random coefficient model or somewhat confusingly because of the generality the heterogeneous treatment effect case. [↑](#footnote-ref-6)
5. Selection bias is a correlation between observed and/or unobserved factors that impact the outcome and the probability of receiving treatment. Essential heterogeneity is a correlation between the treat effect size and the probability of receiving treatment. Therefore, essential heterogeneity is only a possible issue when effects are not homogenous. Put another way, selection bias could be if individuals that would have done well even in absence of treatment adopt the a treatment leading to selection on observables: $CoV(D, X)>0$ or selection on unobservables $Cov(D, \varepsilon)>0$. With essential heterogeneity, these individuals wouldn’t necessarily have done well in absence of treatment, but they benefit more from the treatment when selected into it. [↑](#footnote-ref-7)
